# Development of a model for *Colletotrichum* diseases with calibration for phylogenetic clades on different host plants

**DOI:** 10.3389/fpls.2023.1069092

**Published:** 2023-03-29

**Authors:** Irene Salotti, Yu-Jie Liang, Tao Ji, Vittorio Rossi

**Affiliations:** ^1^ Department of Sustainable Crop Production, Università Cattolica del Sacro Cuore, Piacenza, Italy; ^2^ Department of Agro‐forestry Ecosystems, Universitat Politècnica de València, Valencia, Spain

**Keywords:** epidemiology, disease modeling, glomerella, systematic literature review, model validation

## Abstract

Fungi in the genus *Colletotrichum* cause serious pre- and post-harvest losses to several agricultural crops worldwide. Through a systematic literature review, we retrieved the published information on *Colletotrichum* anthracnose diseases on different host plants and developed a mechanistic model incorporating the main stages of the pathogen’s life cycle and the effect of weather. The model predicts anthracnose progress during the growing season on the aerial organs of different crops, and was parameterized for seven *Colletotrichum* clades (acutatum, dematium, destructivum, gloeosporioides, graminicola, and orbiculare) and the singleton species, *C. coccodes*. The model was evaluated for the anthracnose diseases caused by fungi belonging to five clades on six hosts by using data from 17 epidemics that occurred in Italy, the USA, Canada, and Japan. A comparison of observed versus predicted data showed a concordance correlation coefficient of 0.928 and an average distance between real data and the fitted line of 0.044. After further validation, the model could be used to support decision-making for crop protection.

## 1 Introduction

Fungi in the genus *Colletotrichum* (phylum: Ascomycota, class: Sordariomycetes) cause pre- and post-harvest diseases in horticultural, ornamental, and fruit tree crops worldwide (Hyde et al., 2009; Cannon et al., 2012). The genus *Colletotrichum* was ranked among the top 10 fungal plant pathogens with scientific and economic importance (Dean et al., 2012). This genus is widely distributed in tropical and subtropical regions as well as in temperate and Mediterranean areas. Common hosts range from high-value crops like strawberry, olive, apple, and grape, to staple food crops grown by subsistence farmers in developing countries like sorghum and cassava (Pastor-Corrales and Frederiksen, 1978; Peres et al., 2005; Sangpueak et al., 2018). Many *Colletotrichum* species are primarily reported as causal agents of anthracnose, which is also known by other names, like bitter rot on apple and ripe rot on grape (Dowling et al., 2020). In addition to causing severe yield losses, anthracnose epidemics can reduce the quality of the produce (Waller et al., 2002; Dowling et al., 2020). Blossom blight and fruit rots are often the main economically damaging symptoms; however, necrotic lesions can also appear on leaves, stems, and twigs, leading to the deterioration of plants and a reduction in fruit quality (Dowling et al., 2020).

*Colletotrichum* species are grouped into phylogenetic clades (also called “species complexes”) on the basis of multilocus molecular analysis. Species within a clade are closely related to each other (Cannon et al., 2012; Vieira et al., 2020; Talhinhas and Baroncelli, 2021) and have similar temperature requirements (Salotti et al., 2022). A recent systematic review of the taxonomy and of the phylogenetic clades of the genus identified 15 major clades and a number of small clusters and isolated species (Talhinhas and Baroncelli, 2021). Detailed descriptions of major phylogenetic clades and the list of species in each clade were provided by Cannon et al. (2012); Vieira et al. (2020); Talhinhas and Baroncelli (2021), Damm et al (2009; 2012; 2013; 2014), and Weir et al. (2012).

*Colletotrichum* species are primarily reported as causal agents of anthracnose, occurring on plant leaves, stems, flowers, and fruits. The disease cycles of anthracnose on different hosts have similar components (Peres et al., 2005; De Silva et al., 2017); they are generally polycyclic, with splash-borne asexual spores (conidia) responsible for the initiation and spread of the epidemics. Other *Colletotrichum* diseases have been described, causing crown rot of strawberry and black dot on tomato and potato roots (Dillard and Cobb, 1997; Dillard and Cobb, 1998; Waller et al., 2002). *Colletotrichum* species can also cause post-harvest diseases, especially on avocado, almond, and citrus fruit (Prusky, 1996; Ismail and Zhang, 2004; Prusky et al., 2013); in these cases, the pathogens develop infection structures (appressoria) on the host, but remain dormant inside the fruit until after harvest (i.e., during storage, transportation, or sale of fruit), at which time symptoms develop (Ismail and Zhang, 2004; Prusky et al., 2013). Several studies reported that species within a clade show similar colonization and infection behaviour, while the establishment of quiescence is host- and tissue-specific (Sanders and Korsten, 2003; Damm et al., 2012; Weir et al., 2012; Prusky et al., 2013; Jayawardena et al., 2016; Zakaria, 2021). Species within clades also have similar temperature requirements for mycelial growth, conidial germination and infection, and spore production (Salotti et al., 2022). For example, *C. gloeosporioides*, *C. fragarie*, *C. gossypii*, and *C. musae* belonging to the gloeosporioides clade show optimum temperature for mycelial growth, sporulation, conidial germination and infection between 25 and 32°C (Salotti et al., 2022).

Integrated pest management (IPM) against *Colletotrichum* spp. includes cultural, biological, and chemical control. Cultural control includes producing disease-free propagating materials, avoiding the use of overhead irrigation, limiting rain splashing by mulching, and managing weeds (Coelho et al., 2008; Saxena et al., 2016). The removal of infected crop residues and mummified or rotten fruits is recommended to reduce the inoculum sources and the disease pressure in the field (Samuelian et al., 2012; Saxena et al., 2016). For instance, the removal of crop debris removal reduced by about 30% the anthracnose severity in Ethiopian sorghum fields (Aragaw et al., 2021). The use of resistant cultivars could be an environmentally friendly and cost-effective way to control the disease (Saxena et al., 2016); however, available marketable cultivars often do not provide enough resistance to eliminate the need for fungicides (Dowling et al., 2020). Biological control agents (BCAs), such as *Trichoderma* spp. and *Bacillus subtilus*, have been considered (Saxena et al., 2016; Dowling et al., 2020), but most farmers still prefer combining cultural practices and chemical control methods because of the variable efficacy of BCAs under field conditions (Dowling et al., 2020). Calendar-based application of chemicals, therefore, remains the main tool for controlling anthracnose diseases. This leads to intensive fungicide sprays, which may involve needless applications that are not economically or ecologically sustainable (Rossi et al., 2012).

Mathematical models can help growers schedule fungicide sprays (Pertot et al., 2017; Rossi et al., 2019; Fedele et al., 2022). Models have been developed to understand the epidemiology of anthracnose on specific crops and to support infection-risk-based fungicide applications (Dodd et al., 1991; Park et al., 1992; Monroe et al., 1997; Moral et al., 2012; Singh, 2020). Most of these models, however, are simple and consider only one component of the pathogen life cycle, mainly conidial infection, and only focus on a single *Colletotrichum* species or crop. Furthermore, many of these models have never been validated against independent data (i.e., data not used in model development). Contrary to these simple models, mechanistic models have a flexible structure, a high explanatory ability, and the possibility of making predictions over a wide range of agricultural contexts; as a result, mechanistic models are considered useful for decision-making in IPM (De Wolf and Isard, 2007; Rossi et al., 2019). Recently, Ji et al. (2021) developed and validated a mechanistic, weather-driven model for the ripe rot of grapes caused by the *Colletotrichum* species.

Given that the pathogens causing different *Colletotrichum* diseases affecting the aerial parts of host plants in the field (pre-harvest) have similar life cycles, we developed a general, weather-driven, mechanistic model for anthracnose epidemics. The model is “general” in that it has one conceptual structure that incorporates the key epidemiological components and a set of equations that account for the effect of external variables (both environmental and host-related), which can be calibrated for single *Colletotrichum* species, clades, and host plants. For model development and calibration, we (i) conducted a systematic literature search to retrieve the available information on *Colletotrichum* anthracnose diseases; (ii) used this information to develop a conceptual model of the diseases caused by *Colletotrichum* species based on systems analysis; (iii) developed the mathematical equations describing the system both quantitatively and dynamically; (iv) calibrated the model for seven major clades and the singleton species *C. coccodes* (which is referred to as the coccodes clade in this report); and finally (v) evaluated the ability of the model to represent the real system.

## 2 Materials and methods

### 2.1 Literature search

For developing the conceptual and mathematical structure of the model, we conducted a systematic literature search (Okoli and Schabram, 2010; Biesbroek et al., 2013; Candel, 2014) to collect data on the biology, ecology, and epidemiology of *Colletotrichum* spp. from academic articles, articles in the press, and conference papers. Articles were searched by combining the following keywords: (i) *Colletotrichum*; (ii) conidia OR spore OR acervuli OR primary inoculum OR production OR development OR overwinter OR dissemination OR release OR dispersion OR deposition OR infection OR penetration OR germination OR incubation OR lesion development OR lesion onset OR latency OR latent period, and (iii) temperature OR wetness OR relative humidity OR rain OR environment. Search strings are shown in **Table 1** of the **Supplementary material**. The literature search was carried out in 2022 in three bibliographical databases: Scopus (https://www.scopus.com/ accessed on February 25), Web of Science (https://www.webofscience.com/ accessed on February 28), and CAB Abstracts (https://www.cabdirect.org/cabdirect/search/ accessed on March 1). Each article found was first reviewed on the basis of the information in the title and abstract; articles of potential interest were then read in full. Additional articles were retrieved from the “References” section of the found articles; these articles were also reviewed.

**Table 1:** List of variables, rates, and parameters used in the model, and their units.

Acronym	Description	Unit
State variables		
H	Healthy sites	0 to 1^a^
I	Infectious sites, where visible lesions produce conidia	0 to 1
L	Latent sites, where disease symptoms are not visible	0 to 1
R	Removed sites, where visible lesions are old and non-sporulating	0 to 1
V	Visible sites, where lesions are visible, but non-sporulating yet	0 to 1
Rate variables		
*DISR*	Rate of dispersal of conidia	0 to 1
*INFR*	Rate of infection by conidia	0 to 1
*INCR*	Rate of incubation	0 to 1
*LATR*	Rate of latency	0 to 1
*REMR*	Rate of removal	0 to 1
*SPOR’*	Rate of conidia production on primary inoculum sources	0 to 1
*SPOR”*	Rate of conidia production on secondary inoculum sources	0 to 1
Parameters		
*i*	Duration of infectious period in optimum conditions	N hours
IPmin	Minimum length of the incubation period	N hours
*k*	Abundance of primary inoculum sources	0 to 1
LPmin	Minimum length of the latency period	N hours
*p*	Duration of latency period in optimum conditions	N hours
*r*	Apparent infection rate	N
RcOPT	Optimum Rc value	N
Auxillary variables		
CON	Abundancy of conidia, i.e., CON = CON’ + CON”	N
CON’	Abundancy of conidia on primary inoculum sources	N
CON”	Abundancy of conidia on secondary inoculum sources	N
HS	Susceptibility of the host plant	0/1^b^
IP	Incubation period	N
iP	Infectious period	N
LP	Latency period	N
Computed variables		
CF	Correction factor for diseased sites	0 to 1
D	Diseased sites, i.e., D = L + V + I + R	0 to 1
DS	Disease severity	0 to 1
pi	Hourly progress of IP depending on temperature	N
pl	Hourly progress of LP depending on temperature	N
Rc	Basic infection rate corrected for the removal	N
External variables		
GS	Growth stage of the plant based on the BBCH scale	–
P	Hourly rainfall	mm
T	Hourly air temperature	°C
Teq	Equivalent of temperature	0 to 1
Tmax	Maximum temperature for sporulation	°C
Tmin	Minimum temperature for sporulation	°C
VPD	Vapor pressure deficit	hPa
WD	Duration of a wet period	N hour
Driving functions		
f1(P)	Equation for the effect of rainfall on conidial dispersal and deposition on host tissues	0 to 1
f2(P)	Equation for the effect of rainfall on the wash-off of conidia from host tissues	0 to 1
f(T)	Equation for the effect of temperature on sporulation, or incubation or latency	0 to 1
f(WD)	Equation for the effect of wetness duration on sporulation, or conidial infection	
f’(WD)	First derivative of the equation for the effect of wetness duration on sporulation	0 to 1
RcT	Rc modifier for temperature	0 to 1
RcWD	Rc modifier for wetness duration	0 to 1

a 0 to 1 refers to a dimensionless, continuous variable.

b 0/1 refers to a dimensionless, binomial variable (i.e., 0 = the host is not susceptible to infection; 1 = the host is susceptible to infection).

Selected papers were used to extract information on the influence of environmental conditions on the considered biological processes. Data on the pathogen or the disease were obtained directly from the text, tables, or graphs in the papers; the GetData Graph Digitizer 2.24 (http://getdata-graph-digitizer.com accessed on 5 May 2021) was used to obtain precise data from graphs.

### 2.2 Conceptualization of the system

**Figure 1:**
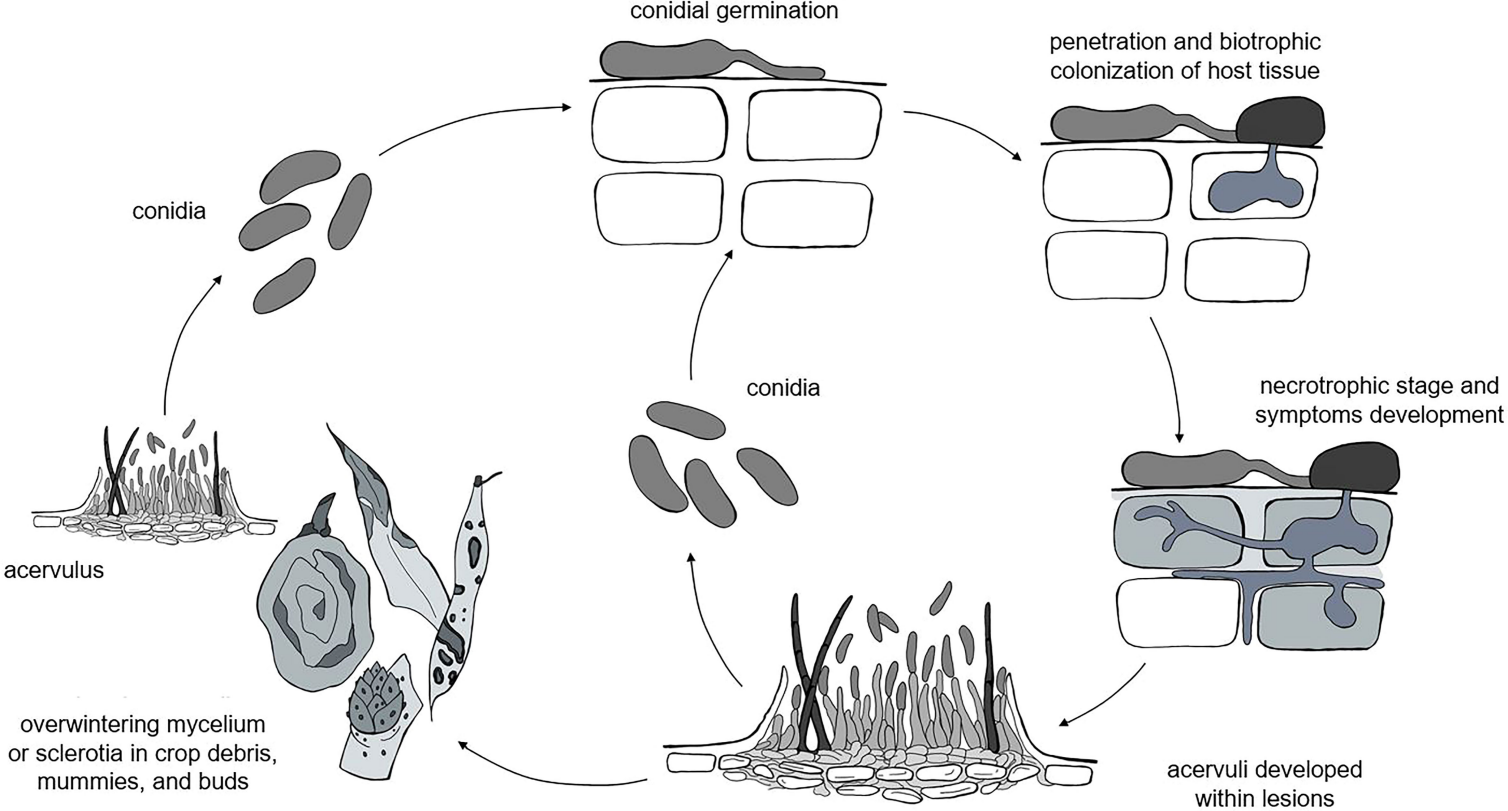
The general life cycle of *Colletotrichum* spp. as considered in the model: the pathogen overwinters as mycelia or sclerotia in different plant residues, which produce acervuli that in turn produce primary conidia. Infections caused by rain-dispersed conidia develop into lesions, which develop acervuli that produce conidia for secondary infection cycles.

Although different life styles have been described for *Colletotrichum* spp. (i.e., necrotrophic, biotrophic, hemibiotrophic, quiescent, and endophytic life styles), the life cycles of these species have some common attributes (Peres et al., 2005; De Silva et al., 2017), which include both sexual and asexual reproduction. Because the perithecial stage is rarely observed in the field (Dowling et al., 2020), the asexual conidia are the most important stage for disease development; our model, therefore, focuses on the asexual life cycle. A general, asexual life cycle for *Colletotrichum* spp. is shown in **Figure 1**.

*Colletotrichum* spp. overwinter as mycelium or sclerotia in crop debris (Farley, 1976; Casela and Frederiksen, 1993; Yoshida et al., 2002; Conner et al., 2019), fruit mummies (Moral and Trapero, 2012; Samuelian et al., 2012; McKay et al., 2014), and buds (Verma et al., 2006; Stensvand et al., 2017; Everett et al., 2018). In some hosts like strawberry and *Citrus* spp., the pathogen survives as melanized appressoria on asymptomatic leaves (Denham and Waller, 1981; Leandro et al., 2001). The pathogen can also be introduced in the field with infected seeds (Thomas and Sweetingham, 2004) or transplants (Howard et al., 1992); infected seeds and transplants, however, are not considered as primary inoculum sources in our model, because we assume that farmers use pathogen-free propagating material.

Primary inoculum consists of fresh conidia produced in acervuli under favorable conditions of temperature (10 to 30°C for most of the clades) and moisture (e.g., wetness periods longer than 6 h) on overwintered sources for the entire cropping season (Moral and Trapero, 2012; Stensvand et al., 2017; Everett et al., 2018). Conidia are splash-dispersed by rain (Denham and Waller, 1981; Chakraborty and Billard, 1995; Madden et al., 1996; Madden, 1997; Guyot et al., 2005; Buirchell et al., 2006), are deposited on plant surfaces, and germinate; the germ tubes then produce appressoria that in turn produce infection pegs that penetrate the host cuticle and epidermal cell walls (Perfect et al., 1999). Penetration through stomata or wounds by germ tubes without the formation of appressoria is rare (Zulfiqar et al., 1996). The infection of epidermal and mesophyll cells occurs after a symptomless biotrophic phase and results in a necrotic phase in which host cells are killed and secondary hyphae grow intra- and inter-cellularly (O'Connell et al., 2012). In some cases, the pathogen has a quiescent phase (Peres et al., 2005; Prusky et al., 2013), and the switch to the necrotrophic phase occurs only when host tissue and environmental conditions become conducive to the continuation of the infection cycle. Except for those few species that exist entirely as endophytes, the majority of *Colletotrichum* species develop a necrotrophic stage at some point in their life cycle (Prusky et al., 2013).

Acervuli finally develop within lesions and produce masses of conidia that serve as secondary inoculum for the repetition of infection cycles and the further spread of the disease (Peres et al., 2005; De Silva et al., 2017).

### 2.3 Model development

**Figure 2:**
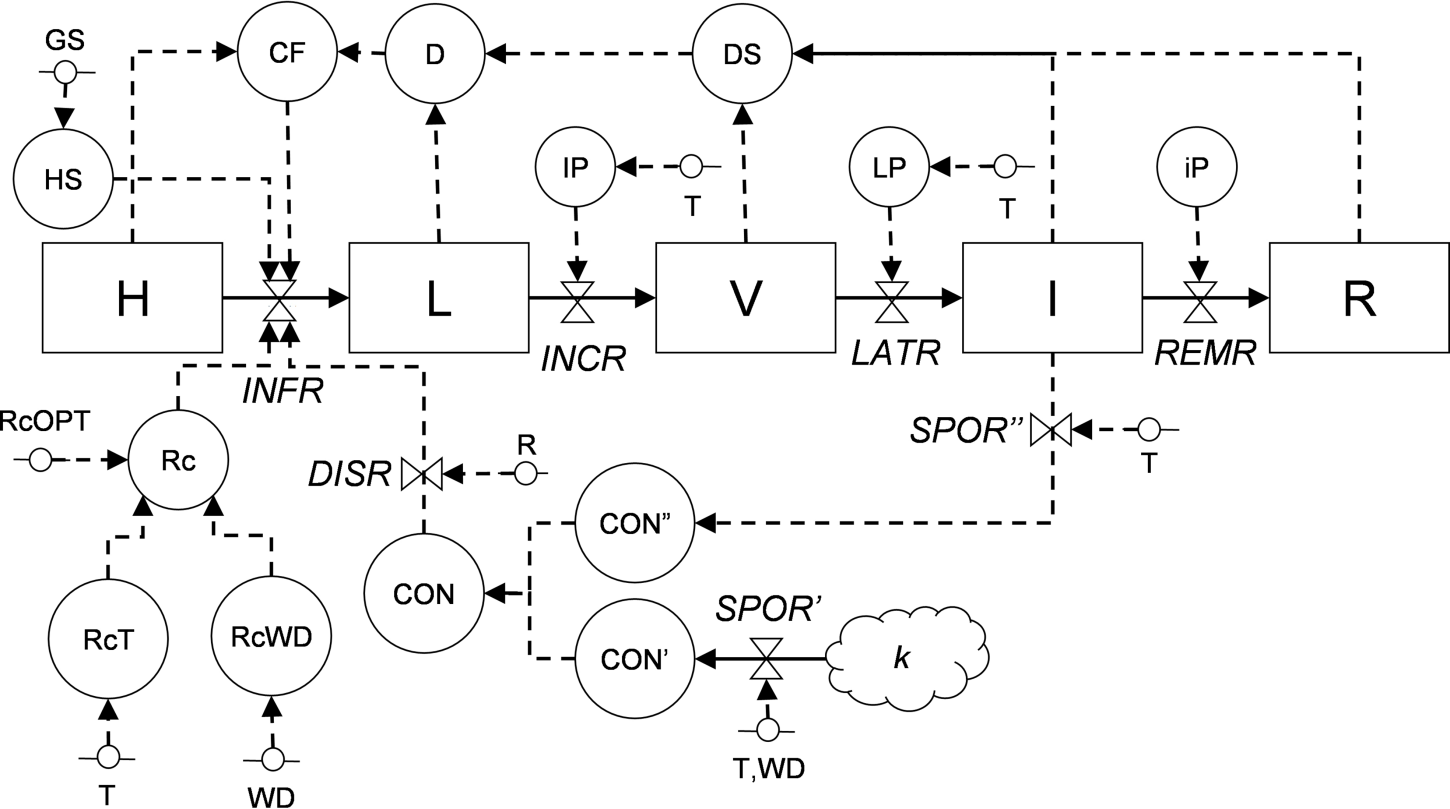
Relational diagram of the processes leading to infection by *Colletotrichum* spp. Acronyms for state variables, rates, and parameters are listed in **Table 1**. The core structure of the model is based on Zadoks (1971), with sites evolving from healthy (H), to latent (L), to visible (V), to infectious (I), and finally to removed (R).

Information from the literature was organized in a relational diagram representing the epidemic structure as developed by Zadoks (1971) and by using the systems analysis syntax (Leffelaar and Ferrari, 1989; Rossi et al., 2010; Rossi et al., 2015) (**Figure 2** and **Table 1**). In this structure, the crop is composed of a large but finite number of sites that have equal dimensions and equal probability of becoming infected; a site is defined as a fraction of the host tissues where an infection may occur and where a lesion may develop (Zadoks, 1971; Savary et al., 2015). During the epidemic, a site goes through the following stages: healthy, infected without visible lesions (latent infection), infected with visible lesions (i.e., disease symptoms or signs), infected with fertile (i.e., sporulating) lesions, and finally, infected with no-longer sporulating lesions (or removed lesions). In the model, the site stages are state variables (boxes); the flow from one stage to the next (solid arrows) is regulated by rates (valves), which are in turn influenced by external and auxiliary variables (short segments and circles, respectively). The external variables include weather variables such as air temperature (T, in °C), wetness duration (WD, in h), and rainfall (P, in mm). Mathematical equations link (through dotted arrows) the external variables to rates, and were developed by using the data extracted from the literature; different parameterization was used for the different phylogenetic clades of *Colletotrichum* (Salotti et al., 2022).

For equation development and parameterization, the original data for each response (independent) variable collected in different experiments were rescaled between 0 and 1 by dividing each value by the maximum value obtained in each experiment. This was necessary because the experiments often used different measurement units (e.g., conidia production has been expressed as the average number of conidia per lesion, the number of conidia per cm^2^ of a lesion, or the number of conidia per Petri dish in laboratory experiments, depending on the paper), fungal species or strains, host plants, etc. As an example of rescaling, Wilson et al. (1990) reported that 4 and 24 h of continuous wetness during infection on strawberry fruits by *C. acutatum* gave a disease incidence of 2 and 100%, respectively; therefore, the rescaled data were x_4h_ = 2/100 = 0.2, and x_24h_ = 100/100 = 1.

Rescaled data were regressed against the influencing environmental factors (the independent variables). Different nonlinear regression models were fit, and the goodness-of-fit was compared based on the Akaike’s Information Criterion (AIC); the model providing the smallest AIC value was considered the most correct (Brunham and Anderson, 2002). Equation parameters were estimated using the function *nls* of the “stats” package of R software (Team, R Core. R: A Language and Environment for Statistical Computing. 2019; available at https://www.r-project.org/). For parameter estimation, the data obtained from different experiments were considered to be replicates; the data concerning the different species belonging to the same clade were also considered to be replicates.

The goodness-of-fit of equations to original (rescaled) data was evaluated based on the adjusted R^2^, the concordance correlation coefficient (CCC), the root mean square error (RMSE), and the coefficient of residual mass (CRM) (Nash and Sutcliffe, 1970; Lin, 1989). The adjusted R^2^ was estimated by conducting a linear regression between the observed values and the model predicted values; the linear regression was conducted with the *lm* function of the R “stats” package (Wickham, 2019). CCC is an indication of the difference between the best fitting line and the perfect agreement line (if CCC = 1, the agreement is perfect) (Lin, 1989). CCC was obtained using the CCC function of the R “DescTools” package (Signorell, 2020). RMSE, which represents the average distance of real data from the fitted line (Nash and Sutcliffe, 1970), was obtained using the *rmse* function of the R “modeler” package (Wickham, 2019). CRM was calculated as described in Manstretta and Rossi (2015); CRM is a measure of the tendency of the equation to overestimate or underestimate the observed values (a negative CRM indicates a tendency of the model toward overestimation; Nash and Sutcliffe, 1970).

The model was developed and run in Excel^®^ (Microsoft 365^®^).

### 2.4 Model evaluation

We evaluated the ability of the model to describe real epidemics by using 17 disease progress curves (or epidemics) obtained from field data and retrieved from the literature. Details on *Colletotrichum* species and clades, primary inoculum origin (natural or artificial), hosts, and experimental sites used for model validation are summarized in **Table 2**.

**Table 2:** Summary description of the 17 epidemics used for model evaluation and source of weather data used to operate the model.

Location year	Year	Host^c^	Period	Epidemic	Weather data^d^
Acutatum clade					
Veglie, Apulia, Italy **(** Anselmi, 2023 **)**	2017	Olive cv. Cellina di Nardo	15 to 30 days Aug 17 – Dec 21	IT-17A^a^	Squinzano station (40°27’N, 18°03’ E); 15 km
Nardò, Apulia, Italy (Anselmi, 2023)	2017		15 days Oct 12 – Dec 21	IT-17B^a^	Sannicola station (40°04’N, 18°04’E); 12 km
Avetrana, Apulia, Italy (Anselmi, 2023)	2018		15 to 30 days Sep 25 – Nov 21	IT-18^a^	Muraggio station (40°19’N; 17°34’E); 16 km
	2019		30 days Sept 23 – Nov 27	IT-19^a^	
Wooster, Ohio, USA (Madden et al., 1993)	1990	Strawberry cv. Tristar	3-4 day interval; Jul 16 – Aug 24	OH-90^b^	Akron Fulton airport (41°02’N, 81°28’W); 35 km
	1991		3 to 4 days Aug 16 – Sep 26	OH-91^b^	
Dematium clade					
Tsukuba, Ibaraki, Japan (Yoshida et al., 2002)	1993	Mulberry	30 days Aug 15 – Oct 15	JA-93^a^	Tsukuba station (36°03’N, 140°07’E); 10 km
	1994		30 days Aug 15 – Nov 15	JA-94^a^	
	1995		30 days Aug 15 – Nov 15	JA-95^a^	
Gloeosporioides clade					
Castle Hayne, North Carolina, USA	1980	Grapevine cv. Carlos	7 days Sep 17 – Oct 1	NC-80^a^	Wilmington airport (34°16’N, 77°54’W); 18 km
(Daykin and Milholland, 1984)	1981		7 days Sep 17 – Oct 1	NC-81^a^	
	1982		15 days Jun 15 – Sep 7	NC-82^a^	
Graminicola clade					
East Lansing, Michigan, USA (Danneberger et al., 1984)	1982	Bluegrass	10 days May 9 – Jul 27	MI-82^a^	Capital Region airport (42°46’N, 84°35’W); 11 km
North Brunswick, New Jersey, USA; (Hempfling et al., 2020)	2009		7 days Jul 31 – Aug 31	NJ-09^a^	Linden airport (40°36’N, 74°14’W); 28 km
	2010		7 days Jul 23 – Aug 12	NJ-10^a^	
Orbiculare clade					
Morden, Manitoba, Canada	2014	Dry bean cv. AC Pintoba	3-4 days Jun 25 – Aug 14	MA-14^b^	Morden station (49°11’N, 98°03’W); 1 km
(Halvorson et al., 2021)	2015		7 days Jun 30 – Aug 14	MA-15^b^	

a The epidemic was started by natural inoculum in the field.

b The epidemic was started by the experimental placement of inoculum in the field or near the field.

c All hosts were susceptible.

d Weather data were downloaded from www.mesonet.agron.iastate.edu; www.data.jma.go.jp; https://www.horta-srl.it

In the validation, we assumed that each epidemic is triggered by a potential quantity of inoculum sources (represented by the parameter *k* in equation [1]), which are responsible for the production of primary conidia. The values of *k* used to initialize the model are reported in **Table 2**. Because there was no information in the literature for an exact calculation of the parameter *k* for each epidemic, the values in **Table 2** were estimated empirically, i.e., we selected the value of *k* that resulted in the closest agreement between predicted and observed disease severities by running the model with varying values of *k* (in the rage 0 to 1).

For epidemics resulting from natural inoculum, the model was operated starting from January 1. For epidemics triggered by the experimental placement of diseased plants (see epidemics MA-14 and MA-15 in **Table 2**) or diseased fruit (see epidemics OH-90 and OH-91 in **Table 2**) in the crop or near the crop, the model was operated starting from the day in which the diseased plants or fruit were placed in the field. For MA-14 and MA-15, the inoculum was introduced by the sowing of *Colletotrichum*-infested white bean seeds (along with non-infested seeds), which developed into plants that produced the primary conidia; the model was operated from June 2 at MA-14 and from June 9 at MA-1. For OH-90 and OH-91, the inoculum was provided by *Colletotrichum*-diseased strawberry fruits that experimenters placed within the crop, and the model was operated from July 16 at OH-90 and from August 7 at OH-91.

Predicted disease severities (i.e., sum of visible, infectious, and removed sites) and observed disease severities were compared, and CCC, RMSE, and CRM (Nash and Sutcliffe, 1970; Lin, 1989), which were used to evaluate the goodness-of-fit of predicted vs. observed data, were calculated by using R software as described before. The different sources of predictive errors in the model were identified by calculating Theil’s partial inequality (Theil’s U statistic) coefficients (Smith and Rose, 1995; Fall et al., 2016); among these coefficients, Ubias indicates the mean difference between observed and predicted disease; Uslope indicates the deviations from the 1:1 line; and Uerror indicates the unexplained variance.

## 3 Results

### 3.1 Model description

The relational diagram of the processes leading to epidemics of *Colletotrichum* spp. is shown in **Figure 2**; abbreviations are explained in **Table 1**. The model is based on host sites, each of which belongs to one of the following mutually exclusive conditions: (i) healthy (H); (ii) latent (L), i.e., without disease symptoms; (iii) visible (V), i.e., with visible lesions; (iv) infectious (I), i.e., with visible lesions producing spores; and (iv) removed (R), i.e., older and non-sporulating lesions removed from the system. The model does not incorporate host growth and senescence and does not account for lesion expansion.

Sites become infected (i.e., they change from healthy sites into latent sites) at an infection rate (*INFR*), which depends on the abundance of conidia (CON), which can be both primary (CON’) and secondary (CON”) conidia, and on their rain-dependent dispersal rate (*DISR*). Primary conidia (produced on *k* overwintered inoculum sources at a sporulation rate of *SPOR’*) and secondary conidia (produced on lesions on the host plant at a sporulation rate of *SPOR”*) accumulate in CON’ and CON”, respectively; *SPOR’* depends on temperature and wetness duration, and *SPOR”* depends only on temperature because the moisture required for sporulation is provided by the host plant. Infection rate also depends on a correction factor (CF) for diseased sites (i.e., stages L, V, I, and R), a multiplication factor (Rc) representing the proportion of daughter lesions generated per mother lesion that depends on temperature (RcT) and wetness duration (RcWD). Infection occurs during the period of host susceptibility (HS), which is regulated by the plant growth stage and varies with the host. Latent sites become visible at the end of an incubation period (IP), and visible sites become infectious at the end of a latency period (LP), both of which depend on temperature; the progress of IP and LP depends on an incubation (*INCR*) and latency (*LATR*) rate, respectively. Infectious sites produce secondary conidia (CON”) during the infectious period (iP) and finally become removed at a removal rate (*REMR*) (**Figure 2**, **Table 1**).

At the beginning of model calculations, H = 1 (i.e., the whole crop is healthy), and the model represents the flow from one state to the following one as a proportion of the H, i.e., on a 0 to 1 scale. The model works at hourly time steps.

#### 3.1.1 Production of primary inoculum

The model considers that the primary inoculum consists of conidia (CON’) produced by *k* inoculum sources at a sporulation rate (*SPOR’*) that depends on temperature and wet periods. On any i^th^ hour, the model calculates *SPOR’* as follows:


(1) SPOR' = k × f(T) × f'(WD)

where the parameter *k* (ranging from 0 to 1) represents the abundance of primary inoculum sources; f(T) accounts for the effect of temperature (T, °C) on the production of conidia; f’(WD) accounts for the effect of wetness duration (WD, hours) on the production of conidia, and is calculated as the first derivative of equation (3). Values of *SPOR’* accumulate in CON’.

The effect of temperature is calculated by a BETE equation (Analytis, 1977) in the following form:


(2) $$f\left(T\right)\;=\;{\left(a\;\times \;Teq^{b}\;\times \;\left(1\;–\;Teq\right)\right)}^{c}$$

where *a* to *c* = equation parameters, and Teq = temperature equivalent in the form Teq = (T–Tmin)/(Tmax–Tmin). For the latter equation, T = hourly temperature, and Tmin and Tmax = minimum and maximum temperature for sporulation, respectively. Tmin, Tmax, and equation parameters of each clade were derived from Salotti et al. (2022).

Whenever there are ≥ 3 continuous hours of wetness, the effect of moisture is calculated as follows (Leandro et al., 2001):

if WD ≥ 3, then


(3) f(WD) = exp (–5.947 × exp (–0.067 × WD))

otherwise, f(WD) = 0.

where WD refers to leaf wetness duration (for the host-pathogen combinations in which the inoculum source is located in the aerial part of the plant, e.g., hanging mummies or bud scales) or ground wetness duration (when the source lays on the ground, e.g., plant debris, leaf litter, or mummies in soil). In the model, the ground is considered wet in hours when rainfall (P) > 0 mm or vapour pressure deficit (VPD) ≤ 4.5 hPa; otherwise, the ground is considered dry (Rossi et al., 2008); VPD is calculated from temperature and relative humidity (RH, in %) following Buck (1981). To calculate WD, wet hours are accumulated until a dry hour occurs; if wetness is restored within 16 h, WD continues to increase until the next dry hour; if the dry period persists for > 16 h, the calculation of WD stops.

Equation (3) was developed and parameterized by fitting the data from Leandro et al. (2003) and Moral and Trapero (2012), which refer to the acutatum clade. Estimates and standard errors of equation parameters were 5.947 ± 1.329 and 0.067 ± 0.009, with adjusted R^2^ = 0.910, CCC = 0.953, RMSE = 0.116, and CRM = 0.041. Because no information was found in the literature on the effect of moisture on sporulation for the other *Colletotrichum* clades, equation (2) was used for all clades.

#### 3.1.2 Dispersal of conidia

The model considers that conidia that accumulated (CON’ for primary conidia and CON” for secondary conidia) are dispersed to host plants at a dispersal rate (*DISR*) that depends on precipitation (P, in mm h^–1^) (Madden et al., 1996; Madden, 1997; Ntahimpera et al., 1999; Guyot et al., 2005), with a minimum of 0.3 mm of rain h^–1^ (Guyot et al., 2005). On any i^th^ hour, the model calculates *DISR* as follows:

if P ≥ 0.3, then


(4) DISR = f1(P) × (1 – f2(P))

otherwise, *DISR* = 0.

where f1(P) accounts for the dispersal of conidia and their deposition on host tissues, and f2(P) accounts for the washing-off of conidia from host tissues due to precipitation intensity > 15 mm h^-1^ (Madden et al., 1996). f1(P) is calculated as follows:


(5) f1(P) = exp (–2.5 × exp(–0.15 × P))

if P > 15, then


(6) f2(P) = 1 / (1 + 966.9 × exp (–0.133 × P))

otherwise, f2(P) = 0.

Equation (5) was developed and parameterized by fitting the data of Madden et al. (1996) and Ntahimpera et al. (1999); estimates and standard errors of equation parameters were 2.5 ± 0.84 and 0.15 ± 0.04, with adjusted R^2^ = 0.895, CCC = 0.953, RMSE = 0.099, and CRM = 0.002. Equation (6) was developed and parameterized by fitting the data of Madden et al. (1996); estimates and standard errors of equation parameters were 966.9 ± 16.4 and 0.133 ± 0.003, with adjusted R^2^ = 0.999, CCC = 0.999, RMSE = 0.004, and CRM = 0.002.

#### 3.1.3 Infection by conidia

Conidia on the plant surface cause infection according to an infection rate (*INFR*), which modulates the transfer of sites from healthy to latent. *INFR* is calculated as follows:


(7) INFR = HS × CON × DISR × Rc × CF

where HS defines whether the host plant is susceptible (HS=1) or not (HS=0); CON is the sum of CON’ and CON”; Rc is a basic infection rate (Vanderplank, 1963); and CF is a correction factor for diseased sites, and is calculated as follows:


(8) CF = 1 – (D / (D + H))

where D is the sum of disease sites (D = L + V + I + R) and H represents healthy sites.

The model accounts for changes in the susceptibility of the host to infection by introducing the correction factor HS into equation (7). HS is defined for each host depending on the species and growth stage. For epidemics occurring on green tissues (leaves, stems, etc.), the model assumes that the plants are susceptible from the appearance of the organ to the end of the season (i.e., harvest for herbaceous hosts; leaf fall for trees and bushes). For epidemics occurring on fruits, HS strictly depends on the reproductive growth stage of the host. For instance, grapevine berries are susceptible from fruit set (BBCH 71; Lorenz et al., 1994) to harvest (BBCH 89; Lorenz et al., 1994; Cosseboom and Hu, 2022), and olive drupes are susceptible from the beginning of flowering (BBCH 61; Sanz-Cortés et al., 2002) to harvest (BBCH 89; Sanz-Cortés et al., 2002; Moral et al., 2009).

In equation (7), Rc represents the proportion of daughter lesions generated per mother lesion. Following Savary et al. (2015), Rc depends on the optimum corrected basic infection rate (RcOPT), which is the basic infection rate under optimum environmental conditions on a susceptible host, and on modifiers for the effect of temperature (RcT) and wetness duration (RcWD) (Loomis and Adams, 1983) as follows:


(9) Rc = RcOPT × RcT × RcWD

The value of RcOPT is estimated following Sun and Zeng (1994) for each *Colletotrichum* clade from disease progress curves as follows:


(10) RcOPT = r / (exp (–r × p) – exp (–r × (i + p)))

where *p* is the latency period under favorable conditions, *i* is the infectious period under favorable conditions, and *r* is the apparent infection rate (Vanderplank, 1963; Vanderplank, 1975) and is calculated as follows:


(11) $$r\;=\;ln\left(x_{2}/\;x_{1}\right)\;/\;\left(t_{2}\;–\;t_{1}\right)$$

where x_1_ and x_2_ are disease fractions on two successive dates (t_1_ and t_2_) at the early stage of the epidemic under conditions conducive to the disease. To calculate *r*, published disease progress curves in susceptible and unprotected crops were used; the first two non-zero severity values (expressed on a 0 to 1 scale) were spotted from these curves and used for the calculation. For each clade, values of RcOPT, *p*, *i*, *r*, and references for the disease progress curves used to calculate *r* are summarized in **Table 3**. The values of *p* were defined based on studies on latency reported in **Table 4**. Irrespective of the clade, the value of *i* was set at 28 days based on King et al. (1997).

**Table 3:** Estimates of the optimum corrected basic infection rate (RcOPT) for eight *Colletotrichum* clades, latency period under favorable conditions for epidemics (*p*), infectious period under favorable conditions for epidemics (*i*), and publications reporting disease progress curves on a susceptible host variety used to calculate the apparent infection rate (*r*).

Clade	RcOPT	*r*	*p*	*i*	References
**acutatum**	0.4	0.16	5	28	Moral and Trapero (2012); McKay et al. (2014); Nekoduka et al. (2018)
**coccodes**	0.2	0.06	14	28	Dillard and Cobb (1997)
**dematium**	0.1	0.05	6	28	Yoshida et al. (2002)
**destructivum**	0.2	0.09	8	28	Chongo et al. (1999)
**gloeosporioides**	0.5	0.19	5	28	Waller (1972); Sweetmore et al. (1994); de Medeiros and Peruch (2012); Berner et al. (2014)
**graminicola**	0.3	0.13	7	28	Li and TeBeest (2009); Moore et al. (2010); Hempfling et al. (2015)
**orbiculare**	0.3	0.12	7	28	Ntahimpera et al. (1996); Kumar et al. (1999)
**truncatum**	0.1	0.02	5	28	Saha and Bera (2021)

RcOPT and *r* were estimated following Sun and Zeng (1994). Values of *p* were defined based on studies on latency reported in **Table 5**. Irrespective of the clade, the value of *i* was set at 28 days based on King et al. (1997).

**Table 4:** Cardinal temperatures, estimates of the optimum temperatures with their standard error, estimates of the shortest duration of incubation (IPmin) and latency (LPmin) at optimum temperature with their standard error, and goodness-of-fit of equation (16) for incubation and equation (17) for latency for each clade.

Clade		Tmin	Topt	Tmax	R^2^	CCC	CRM	References
**Incubation**	**IPmin**							
acutatum	118.3 ± 5.8	5.0	24.2 ± 0.5	30.5	0.930	0.952	–0.024	Gonçalves et al. (2021); Moreira et al (2021; 2020); King et al. (1997); Bertetti et al. (2009); Diéguez-Uribeondo et al. (2011); Greer et al. (2011); Lima et al. (2011); Kenny et al. (2012); Uematsu et al. (2012); Miles et al. (2013); Soares-Colletti and Lourenço (2014); Rodriguez-Salamanca et al. (2015); Everett et al. (2018); López-Moral et al. (2019)
coccodes	157.9 ± 37.2	7.0	28.0 ± 0.5	36.0	0.860	0.761	–0.058	Dillard (1989); Hong and Hwang (1998); Rodriguez-Salamanca et al. (2018)
destructivum	81.8 ± 10.6	5.0	29.1 ± 2.9	36.0	0.854	0.925	0.027	Chongo and Bernier (2000)
gloeosporioides	80.3 ± 10.4	2.0	28.0 ± 2.5	40.0	0.890	0.933	0.091	Gonçalves et al. (2021); Moreira et al (2021; 2020); Ling and Yang (1944); King et al. (1997); de Bellaire et al. (2000); Lim et al. (2002); Monteiro et al. (2009); Wang et al. (2010); Greer et al. (2011); Lima et al. (2011); Kenny et al. (2012); Guyader et al. (2013); Soares-Colletti and Lourenço (2014); Wang et al. (2015); Casanova et al. (2017)
graminicola	72.0 ± 4.8	10.0	30.6 ± 1.2	35.0	0.924	0.869	0.002	Leonard and Thompson (1976); Yang et al. (2000); Khan and Hsiang (2003)
orbiculare	112.3 ± 5.3	6.0	25.1 ± 0.6	33.0	0.90	0.924	0.039	Monteith (1928); Thompson and Jenkins (1985); Dalla Pria et al. (2003)
truncatum	115.7 ± 8.4	5.0	28.2 ± 1.7	40.0	0.699	0.850	0.009	Hartman and Wang (1992); Datar (1995); Hingole et al. (2011); Kumar (2012); Diao et al. (2014); Bi et al. (2017)
**Latency**	**LPmin**							
acutatum	140.4 ± 27.0	2.0	28.8 ± 3.6	40.0	0.848	0.927	0.047	Magalhaes et al. (2021); Moreira et al (2021; 2020); King et al. (1997); Diggle et al. (2002); Kenny et al. (2012); Moral et al. (2012); Soares-Colletti and Lourenço (2014); Han et al. (2016)
coccodes	301.0 ± 12.2	10.0	24.3 ± 0.7	40.0	0.927	0.966	0.007	Rodriguez-Salamanca et al. (2018);
destructivum	202.0 ± 14.5	4.0	27.1 ± 2.1	37.0	0.790	0.896	0.003	Chongo and Bernier (2000)
gloeosporioides	105.7 ± 10.1	2.0	25.3 ± 1.3	36.0	0.755	0.847	0.104	Moreira et al. (2021; 2020); King et al. (1997); Monteiro et al. (2009); Kenny et al. (2012); Guyader et al. (2013); Soares-Colletti and Lourenço (2014); Han et al. (2016)
graminicola	96.0 ± 24.0	10.0	30.6 ± 1.2	35.0	0.924	0.869	0.002	Leonard and Thompson (1976); Yang et al. (2000); Khan and Hsiang (2003)
orbiculare	164.3 ± 24.0	6.0	25.1 ± 0.6	33.0	0.90	0.924	0.039	Monteith (1928); Thompson and Jenkins (1985); Dalla Pria et al. (2003)
truncatum	122.3 ± 9.5	12.0	23.7 ± 0.5	40.0	0.903	0.943	0.030	Datar (1995); Kumar (2012)

The modifier RcT accounts for the effect of air temperature on infection, and is calculated as a BETE equation (Analytis, 1977) in the form of equation (3); Tmin and Tmax for infection by conidia and equation parameters were derived from Salotti et al. (2022) for each clade.

The modifier RcWD accounts for the effect of leaf wetness duration and is calculated on any i^th^ hour as the first derivative of equations (12) to (15), depending on the *Colletotrichum* clade.

For the acutatum clade, equation (12) was developed and parametreized by fitting the data of Wilson et al. (1990); Turechek et al. (2006); Gillett and Schilder (2009); Bertetti et al. (2009); Diéguez-Uribeondo et al. (2011); Moral et al. (2012); Miles et al. (2013); Greer et al. (2014); Rodriguez-Salamanca et al. (2015), and Gonçalves et al. (2021). For the coccodes clade, equation (12) was developed and parameterized by fitting the data of Andersen and Walker (1985); Dillard (1989); Byrne et al. (1998); Hong and Hwang (1998); Sanogo et al. (2003), and Rodriguez-Salamanca et al. (2018):


(12) f(WD) = exp (–d × exp(–e × WD))

For the acutatum clade, equation parameters and their standard errors were *d* = 5.491 ± 0.232, *e* = 0.207 ± 0.042, with adjusted R^2^ = 0.894, CCC = 0.949, RMSE = 0.115, and CRM = –0.027. For the coccodes clade, equation parameters and their standard errors were *d* = 7.343 ± 0.696, *e* = 0.164 ± 0.028, with adjusted R^2^ = 0.956, CCC = 0.979, RMSE = 0.083, and CRM = 0.008.

For the dematium clade, equation (13) was developed and parameterized by fitting the data of Uysal and Kurt (2017):


(13) f(WD) = 1 – exp (–0.1 × WD)

The equation parameter and its standard error were 0.10 ± 0.02, with R^2^ = 0.865, CCC = 0.943, RMSE = 0.09, and CRM = –0.004.

For the destructivum clade, equation (14) was developed and parametreized by fitting the data of Chongo and Bernier (2000). For the gloeosporioides clade, equation (14) was developed and parameterized by fitting the data of Griffin et al. (1987); Chakraborty et al. (1990); Yun and Park (1990); Hildebrand and Jensen (1991); Pandey et al. (1997); Luo and TeBeest (1999); Monteiro et al. (2009); Guyader et al. (2013); Wang et al. (2015); Zhao et al. (2020), and Gonçalves et al. (2021). For the orbiculare clade, equation (14) was developed and parametreized by fitting the data of Auld et al. (1989); Monroe et al. (1997), and Dalla Pria et al. (2003):


(14) $$f\left(WD\right)\;=\;{\left(1\;–\;f\;\times \;exp\;\left(–g\;\times \;WD\right)\right)}^{\left(1\;/\;\left(1\;-\;h\right)\right)}$$

For destructivum clade, equation parameters and their standard errors were *f* = 1.0 ± 0.274, *g* = 0.201 ± 0.157, and *h* = 0.635 ± 0.162, with adjusted R^2^ = 0.885, CCC = 0.952, RMSE = 0.105, and CRM = 0.024. For gloeosporioides clade, equation parameters and their standard errors were *f* = 1.0 ± 0.167, *g* = 0.157 ± 0.039, and *h* = 0.8 ± 0.064, with adjusted R^2^ = 0.939, CCC = 0.968, RMSE = 0.098, and CRM = 0.041. For orbiculare clade, equation parameters and their standard errors were *f* = 0.9 ± 0.157, *g* = 0.168 ± 0.061, and *h* = 0.738 ± 0.083, with adjusted R^2^ = 0.904, CCC = 0.954, RMSE = 0.107, and CRM = 0.019.

For the truncatum clade, equation (15) was developed and parameterized by fitting the data of Singh et al. (2017):


(15) f(WD) = 1 / (1 + 50.0 × exp (–0.289 × WD))

Equation parameters and their standard errors were 50.0 ± 2.473 and 0.289 ± 0.039, with adjusted R^2^ = 0.988, CCC = 0.989, RMSE = 0.057, and CRM = –0.041.

No information on the effect of WD on infection was retrieved for the graminicola clade. The model assumes that there is a similarity between the infection pattern of the graminicola and destructivum clades on the basis of the inter-clade variability studies of Salotti et al. (2022); the model therefore calculates RcWD for the graminicola clade by using equation (14) as parameterized for the destructivum clade.

#### 3.1.4 Incubation and latency periods

Sites flow from L to V at an incubation rate (INCR, which depends on IP, i.e., the incubation period), and then from V to I with a latency rate (LATR, which depends on LP, i.e., the latency period). In each i^th^ hour, the model calculates the hourly progress of both IP and LP as a function of temperature by using the equation of Magarey et al. (2005) in the following form:


(16) pi = f(T) / IPmin

(17) pl = f(T) / LPmin

where pi and pl represent the progression of incubation and latency, respectively, on any i^th^ hour; IPmin and LPmin are the shortest duration of incubation and latency at the optimum temperature; and f(T) is the equation accounting for the influence of temperature. f(T) is calculated as follows:


(18) $$f\left(T\right)\;=\;\frac{\left(T\;-\;Tmin\right)}{\left(Topt\;-\;Tmin\right)}\;\times \;\left(\;\frac{\left(Tmax\;-\;T\right)}{\left(Tmax\;-\;Topt\right)}\;\right){\;}^{\frac{\left(Tmax\;-\;Topt\right)}{\left(Topt\;-\;Tmin\right)}}\;$$

where Tmin and Tmax = minimal and maximal temperature for incubation or latency, respectively; when T< Tmin or T > Tmax, f(T) = 0. Equation parameters are shown for each clade in **Table 4**. No information was available in the literature for the dematium clade; based on between-clade similarities in temperature requirements shown by Salotti et al. (2022), parameters estimated for the acutatum clade were applied to the dematium clade.

The model accumulates the hourly progress of incubation and latency beginning with the hour when the infection has occurred; when the sum of hourly progress results in IP ≥1, *INCR* = 1 and sites flow from L to V; when the sum of hourly progress results in LP ≥1, *LATR* = 1 and sites flow from V to I.

#### 3.1.5 Infectious period and secondary inoculum production

Infectious sites (I) continue producing conidia for an infection period (iP), and then flow from I to R according to a removal rate (*REMR*). The model assumes that fertile lesions continue to produce conidia for the entire epidemic, so that *REMR* = 0. The I sites produce secondary conidia at a sporulation rate (*SPOR”*) that depends on temperature according to equation (2); the secondary conidia accumulate in CON”. Based on King et al. (1997), the model assumes that the pathogen obtains sufficient moisture from the host to produce conidia and does not require free surface moisture for sporulation.

The contribution of secondary inoculum to the progress of the epidemic has been proved for some host-pathogen combinations (Fitzell and Peak, 1984; Smith, 2008; Everett et al., 2018), but not for others. In olive orchards, for instance, mummies are the main source of inoculum all season long, and the contribution of conidia produced within fruit lesions is negligible (Moral et al., 2012); when lesions do not contribute to the progress of the epidemic, CON” = 0.

#### 3.1.6 Predicted disease severity

The model calculates disease severity (DS) during the epidemic as the sum of the proportion of sites with disease symptoms, i.e., visible, infectious, and removed sites, as follows:


(19) DS = V+ I + R

### 3.2 Model evaluation

The model was validated for the acutatum, dematium, gloeosporioides, graminicola, and orbiculare clades. The model’s ability to predict disease development throughout the season was evaluated for 17 epidemics (**Table 2**) recorded between 1980 and 2019 in Italy, the USA, Canada, and Japan on six hosts, i.e., olive, strawberry, mulberry, grape, bluegrass, and dry bean. In this manuscript, only a few epidemics are described in detail; details on the remaining epidemics are provided in the **Supplementary material**.

**Figure 3:**
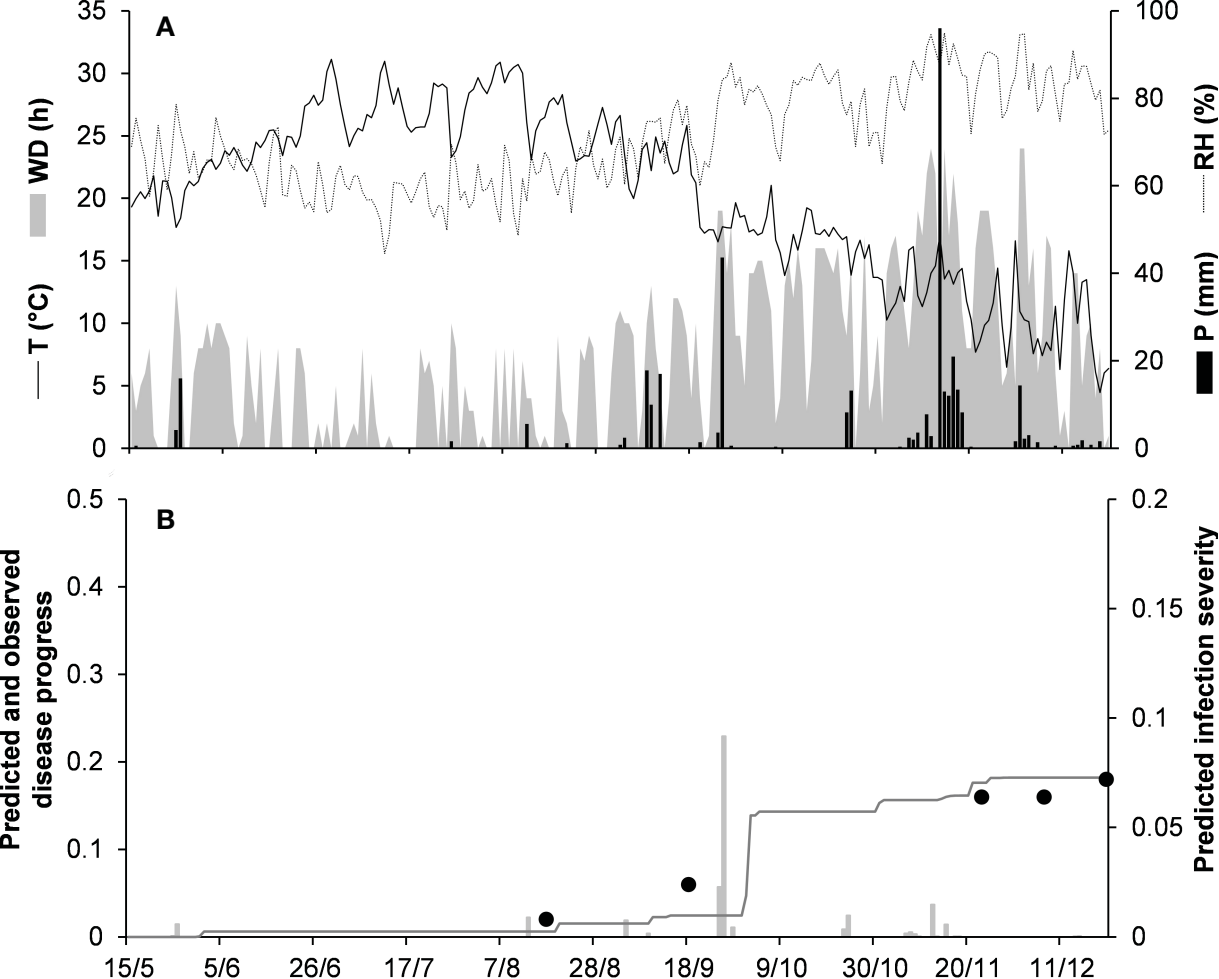
Predicted and observed disease progress on olive (susceptible cv. Cellina di Nardo) for the acutatum clade in Veglie, Apulia, Italy, in 2017 (IT-17A). **(A)** Weather variables: air temperature (T, °C, solid line), relative humidity (RH, %, dotted line), rainfall (P, mm, black bars), and wetness duration (WD, in h, gray area). **(B)** Infection severity predicted by the model (light gray bars), disease severity predicted by the model (dark gray line), and observed disease incidence (full dots).

For the acutatum clade, model validation was performed for 4 epidemics on drupes recorded in naturally infested olive orchards in South Italy, and 2 epidemics in artificially infested strawberry fields in Ohio, USA; in the latter case, researchers provided the inoculum by placing affected fruit between the strawberry rows. Epidemics on olive developed between August and December, with final disease incidence on drupes ranging from 7% to 26% (**Table 5**); CCC between predicted and observed disease ranged from 0.789 to 0.953, and RMSE ranged from 0.022 to 0.047. Epidemics in the two strawberry fields occurred in July to August in OH-90, and in August to September in OH-91, with final disease incidences of 28% and 45%, respectively; in the comparison of predicted and observed disease, CCC = 0.874 and 0.898, and RMSE = 0.055 and 0.061, respectively (**Table 5**). Across all 6 epidemics of the acutatum clade, CCC = 0.895, RMSE = 0.048, and CRM = −0.099 (the latter indicated a slight tendency of the model toward overestimation). An example of model output for acutatum clade is shown in **Figure 3** for IT-17A. In the olive orchard, flowering (BBCH 61, i.e., the beginning of host susceptibility) began on May 15, and harvest occurred at the end of December. From mid-May to the end of December, the average daily temperature was 20.3°C (min = 4.5°C, max = 31.1°C), with an average RH = 71%, a total of 365 mm of rain on 69 rainy days, and a total of 1617 h of leaf wetness (**Figure 3A**). Rains were frequent and intense between September and November, with prolonged wetness periods that led to the prediction of repeated infection periods (**Figure 3B**). Disease outbreak occurred on August 17 (disease incidence 2%) and was followed by a regular disease increase that resulted in a final disease incidence of 18%, which was correctly predicted by the model (**Figure 3B**). For IT-17A, CCC = 0.953, RMSE = 0.022, and CRM = 0.017 (the latter indicated no substantial underestimation).

**Table 5:** Epidemics considered for model validation.

Epidemic	Final disease	*k*	N	CCC	RMSE	CRM
IT-17A	18^a^	0.50	5	0.953	0.022	0.017
IT-17B	7^a^	0.18	6	0.834	0.023	0.379
IT-18	26^a^	0.45	4	0.852	0.047	–0.034
IT-19	15^a^	0.09	3	0.789	0.023	0.111
OH-90	28^b^	0.004	10	0.874	0.055	–0.159
OH-91	45^b^	0.004	13	0.898	0.061	–0.115
Overall results for the 6 acutatum clades above				0.895	0.048	–0.099
JA-93	13^a^	0.00004	3	0.966	0.014	0.095
JA-94	37^a^	0.00004	4	0.991	0.020	0.078
JA-95	23^a^	0.00004	4	0.862	0.055	–0.257
Overall results for the 3 dematium clades above				0.950	0.036	–0.023
NC-80	54^a^	0.003	3	0.843	0.101	–0.161
NC-81	24^a^	0.0002	3	0.497	0.071	–0.152
NC-82	24^a^	0.0002	7	0.993	0.012	0.120
Overall results for the 3 gloeosporioides clades above				0.910	0.068	–0.109
MI-82	25^b^	0.075	9	0.997	0.006	0.050
NJ-09	16^b^	0.085	5	0.495	0.037	0.190
NJ-10	41^b^	0.066	4	0.970	0.034	–0.147
Overall results for the 3 graminicola clades above				0.973	0.026	0.018
MA-14	<1^b^	–	5	–	–	–
MA-15	20^b^	0.08	7	0.896	0.035	0.190
Overall results for the 2 orbiculare clades above				0.931	0.027	0.158
Overall results for all 17 clades				0.928	0.044	–0.052

a % of disease severity

b % of disease incidence Epidemics are labeled for location and year for each clade; the value of final disease incidence or severity; the value of *k* used to parameterize the run; the number of field observations (N); and parameters indicating model performance in predicting the epidemic.

**Figure 4:**
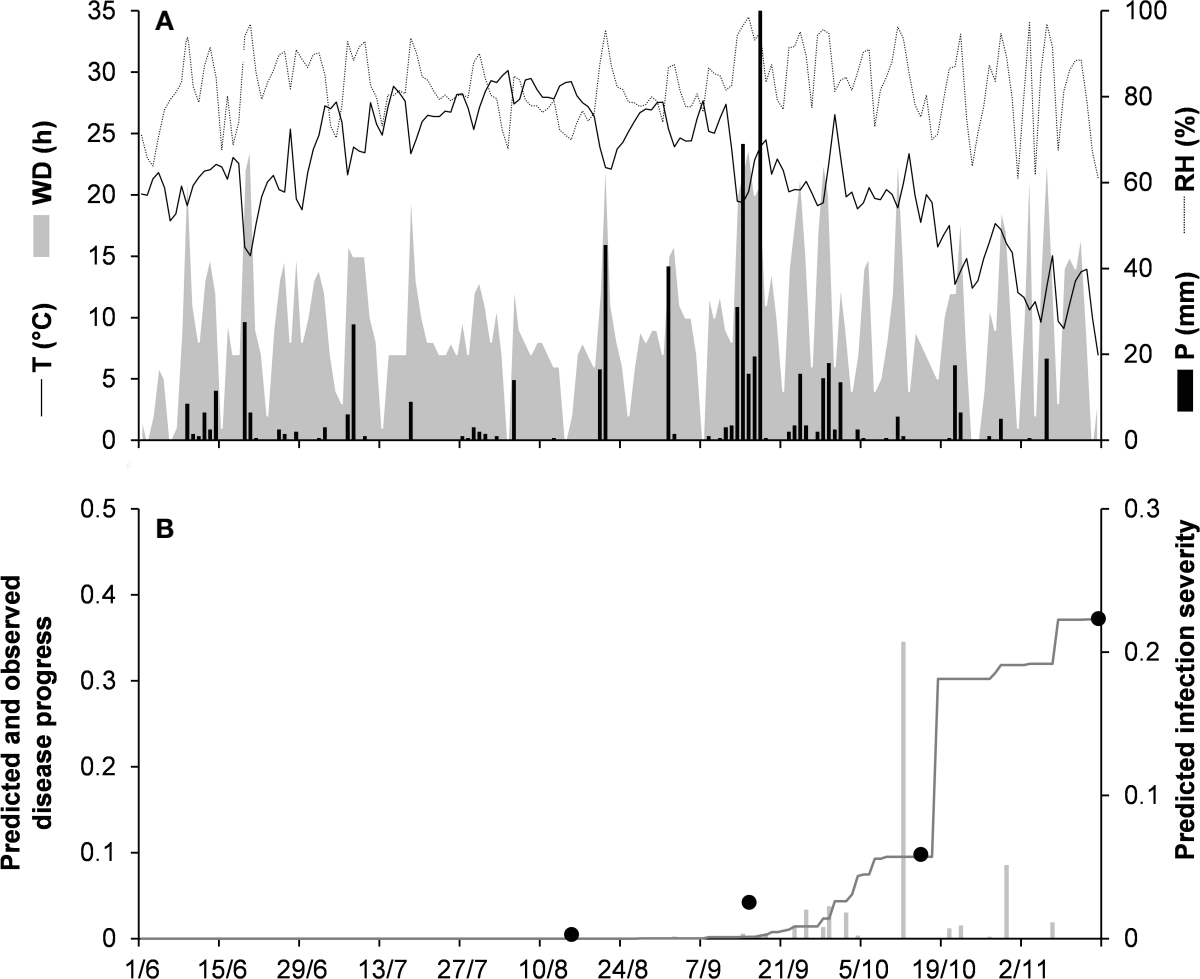
Predicted and observed disease progress on mulberry for the dematium clade in Tsukuba, Ibaraki, Japan, in 1994 (JA-94). **(A)** Weather variables: air temperature (T, °C, solid line), relative humidity (RH, %, dotted line), rainfall (P, mm, black bars), and wetness duration (WD, in h, gray area). **(B)** Infection severity predicted by the model (light gray bars), disease severity predicted by the model (dark gray line), and observed disease incidence (full dots).

For the dematium clade, 3 disease progress curves on mulberry in Japan were used to validate the model (**Table 2**). Epidemics developed on mulberry leaves between August and November, with final disease incidence ranging from 13% to 37%. In the comparison of observed and predicted disease progress curves, CCC ranged from 0.862 to 0.991 and RMSE ranged from 0.014 to 0.055; across all three epidemics, CCC = 0.95, RMSE = 0.036, and CRM = −0.023 (the latter indicated a slight tendency toward overestimation (**Table 5**). An example of model output for the dematium clade is shown in **Figure 4** for JA-94. Disease assessment began in June after the summer-pruning of mulberry trees, and ended in November. During this period, the average daily temperature was 21.9°C (min = 7.0°C, max = 30.1°C), with an average RH = 82%, a total of 625.5 mm of rain on 61 rainy days, and a total of 1688 h of leaf wetness (**Figure 4A**). Regularly distributed rainfall ensured that the epidemic would progress during the season, starting from the time of disease onset in August until November when a sharp increase in disease led to a 37% disease incidence (**Figure 4B**). The model correctly predicted this dynamic; across all three epidemics, CCC = 0.991, RMSE = 0.02, and CRM = 0.078.

**Figure 5:**
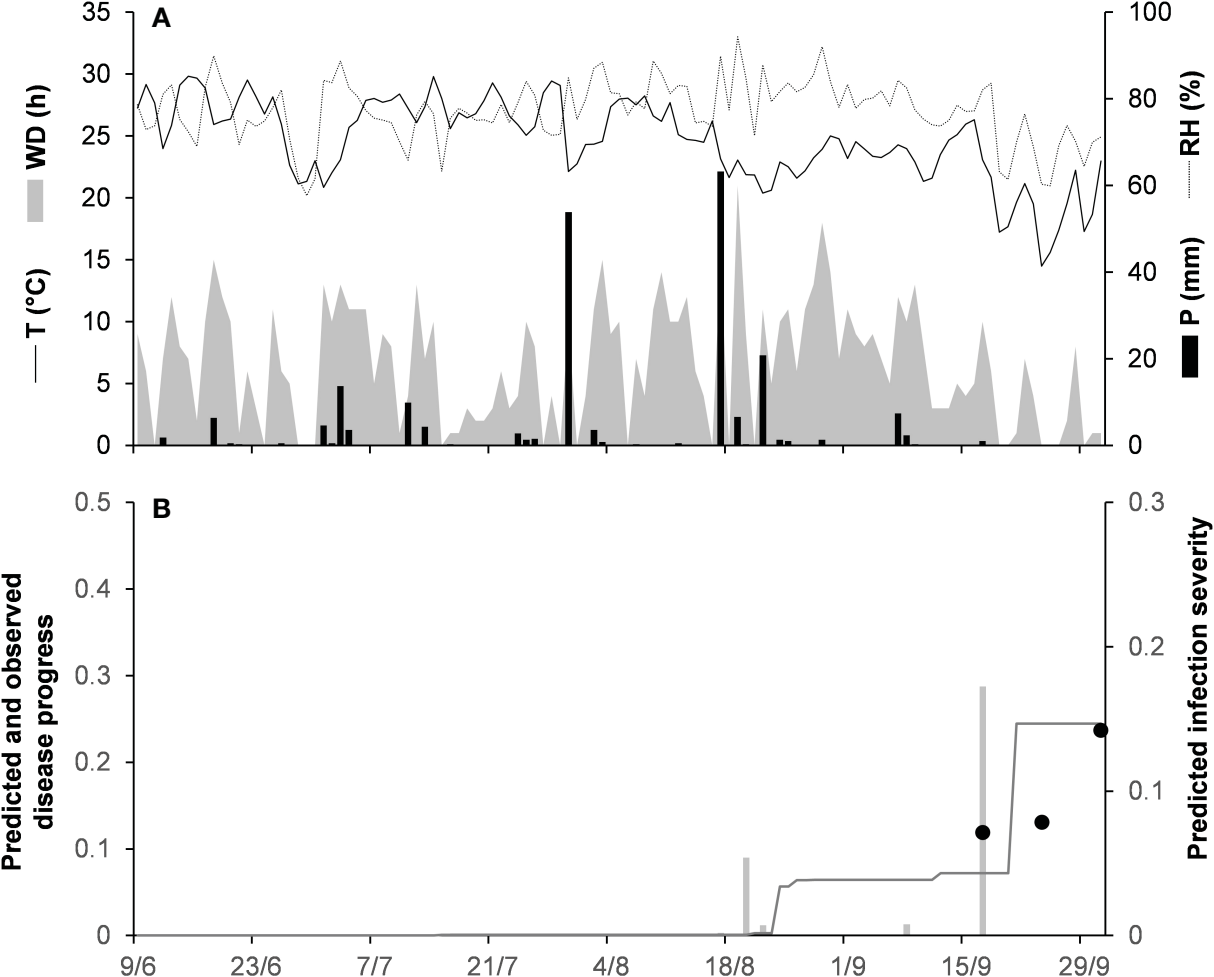
Predicted and observed disease progress on grape for the gloeosporioides clade in Castle Hayne, North Carolina, USA, in 1981 (NC-81). **(A)** Weather variables: air temperature (T, °C, solid line), relative humidity (RH, %, dotted line), rainfall (P, mm, black bars), and wetness duration (WD, in h, gray area). **(B)** Infection severity predicted by the model (light gray bars), disease severity predicted by the model (dark gray line), and observed disease incidence (full dots).

Model validation for the gloeosporioides clade was performed for 3 epidemics on grapevines in North Carolina, USA (**Table 2**). These epidemics developed between June and September, with final disease incidence on berries ranging from 24% to 54%. A small average distance of real data from the fitted line was observed for the three epidemics, with RMSE ranging from 0.012 to 0.101. Concordance between observed and predicted values for NC-80 and NC-82 gave CCC = 0.843 and 0.993, respectively. The NC-81 epidemic is reported in **Figure 5**. Berries were susceptible between June 9 (BBCH 71, i.e., fruit set) and October 1 (BBCH 89, i.e., berries ripe for harvest). During this period, the average daily temperature was 24.6°C (min = 14.5°C, max = 29.8°C), with an average RH = 77%, a total of 216.1 mm of rain on 31 rainy days, and a total of 746 h of leaf wetness (**Figure 5A**). Disease incidence on berries was assessed on September 17 and 24, and on October 1, with a final disease incidence of 24%. Contrary to observations, the model anticipated by some days the observed increase in disease at the end of the season (**Figure 5B**), and CCC = 0.497, probably because of an imprecise estimation of the incubation length in relation to the berry growth stage at the time of infection. In the comparison of predicted and observed disease progress curves for the 3 epidemics of the gloeosporioides clade, CCC = 0.910, RMSE = 0.068, and CRM = −0.109 (the latter indicated a slight tendency of the model toward overestimation).

**Figure 6:**
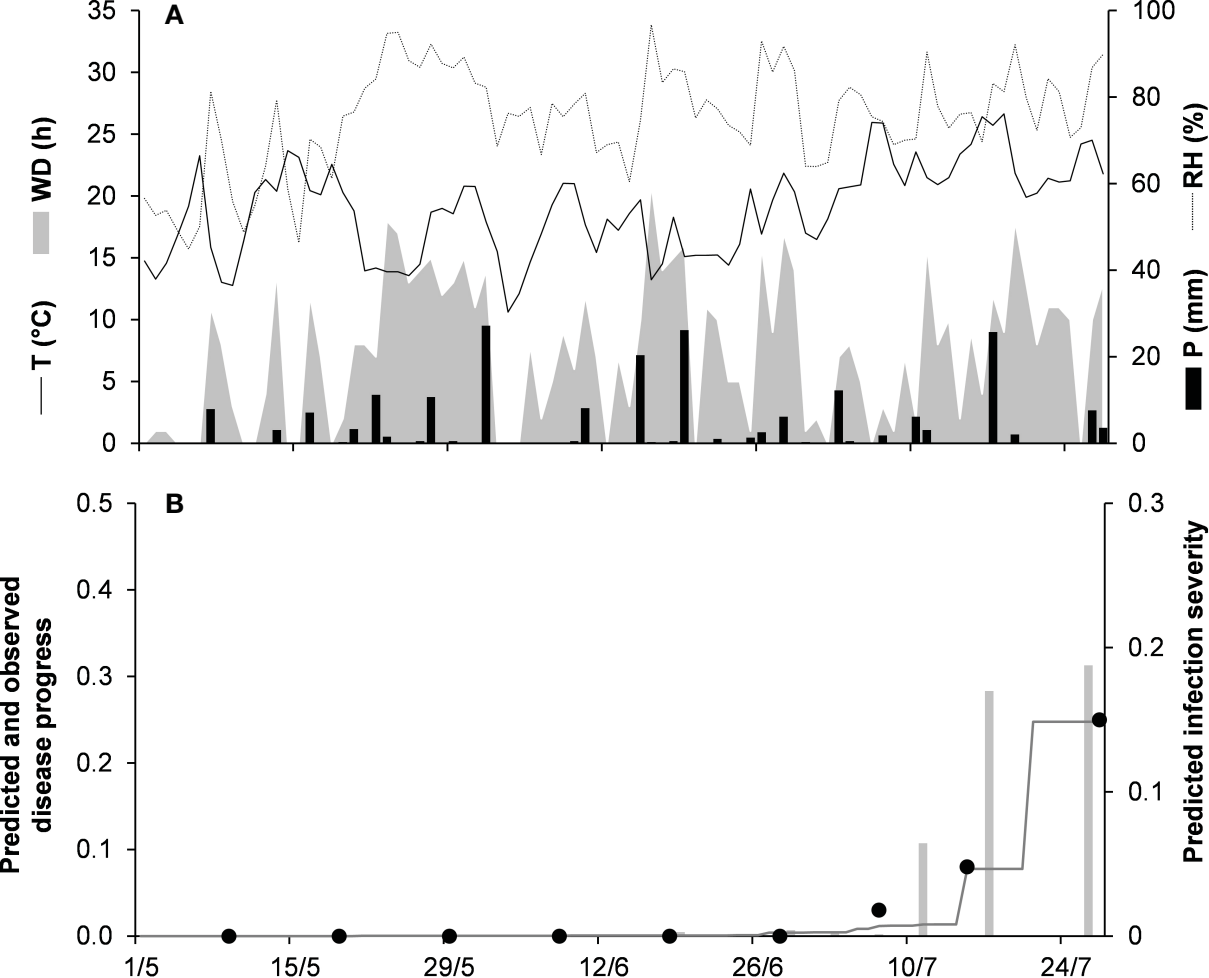
Predicted and observed disease progress on bluegrass for the graminicola clade in East Lansing, Michigan, USA, in 1982 (MI-82). **(A)** Weather variables: air temperature (T, °C, solid line), relative humidity (RH, %, dotted line), rainfall (P, mm, black bars), and wetness duration (WD, in h, gray area). **(B)** Infection severity predicted by the model (light gray bars), disease severity predicted by the model (dark gray line), and observed disease incidence (full dots).

Model validation for the graminicola clade was performed for 3 bluegrass epidemics in New Jersey, USA, in 2009 and 2010, and Michigan, USA, in 1982 (**Table 2**). The epidemics were observed between May and August, with final disease severity ranging from 16% to 41%. In the comparison of model output and observed data, CCC ranged from 0.947 to 0.997, RMSE ranged from 0.006 to 0.037 (**Table 5**), and CRM ranged from −0.15 to 0.19 (the latter indicated a slight over- or underestimation of observed disease progress curves, depending on the epidemic). Across all three epidemics of the graminicola clade, CCC = 0.973, RMSE = 0.026, and CRM = 0.018 (the latter indicated a slight tendency of the model toward underestimation). An example of model output for the graminicola clade is shown in **Figure 6**. At MI-82, the bluegrass field was inspected at 10-day intervals for anthracnose symptoms from the beginning of May to the end of July. During this period, the average daily temperature was 18.9°C (min = 10.6°C, max = 26.7°C), with an average RH = 75%, a total of 202.6 mm of rain on 31 rainy days, and a total of 651 h of leaf wetness (**Figure 6A**). The disease outbreak at MI-82 occurred on July 7 (disease severity 3%), and the disease increased regularly to a final disease incidence of 25%, which was correctly predicted by the model (**Figure 6B**), with CCC = 0.997, RMSE = 0.006, and CRM = 0.050 (the latter indicated a slight tendency of the model toward underestimation).

**Figure 7:**
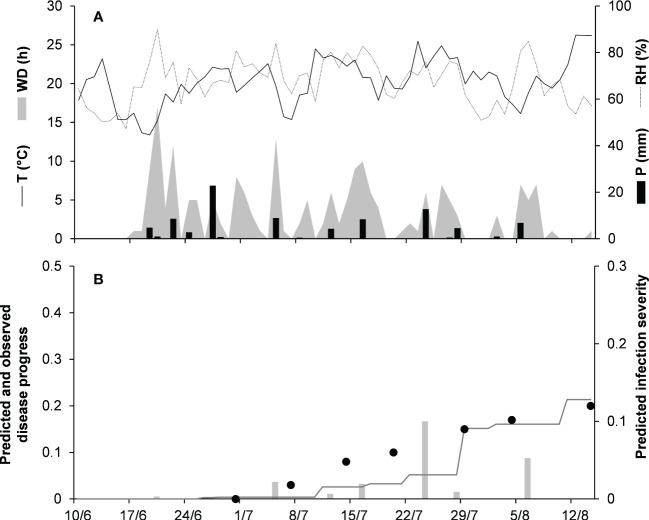
Predicted and observed disease progress on dry bean for the orbiculare clade in Morden, Manitoba, Canada, in 2015 (MA-15). **(A)** Weather variables: air temperature (T, °C, solid line), relative humidity (RH, %, dotted line), rainfall (P, mm, black bars), and wetness duration (WD, in h, gray area). **(B)** Infection severity predicted by the model (light gray bars), disease severity predicted by the model (dark gray line), and observed disease incidence (full dots).

To validate the model for the orbiculare clade, 2 epidemics that occurred on white bean in Manitoba, Canada were used (**Table 2**). Crops were scouted for disease from the end of June, when artificial inoculum was introduced into the field, to mid-August. At MA-14, the disease was recorded only in traces (<1%), so that proper model evaluation was impossible. At MA-15 (**Figure 7**), the average daily temperature was 18.9°C (min = 10.6°C, max = 26.7°C), with an average RH = 75%, a total of 202.6 mm of rain on 31 rainy days, and a total of 651 h of leaf wetness (**Figure 7A**). Regularly distributed rainfalls and prolonged wet periods ensured a progressive development of the epidemic from the first third of July until mid-August, with a final disease severity of 20%. The model slightly overestimated the disease dynamics in the second half of July (**Figure 7B**), with CCC = 0.896, RMSE = 0.035, and CRM = 0.19 (the latter indicated a tendency of the model toward underestimation). Across both orbiculare clade epidemics, CCC = 0.931, RMSE = 0.027, and CRM = 0.158 (the latter indicated a tendency of the model toward underestimation).

In an overall comparison of predicted versus observed values for the 17 epidemics listed in **Table 5**, CCC = 0.928 and RMSE = 0.044; the similarity between the observed data and the fitted line indicated that the model accurately represented the mechanisms leading to *Colletotrichum* epidemics on different host plants. The model, however, showed a slight tendency toward overestimation (CRM = −0.052) when evaluated for the epidemics on different host plants. The variance explained by the relationship of observed versus predicted data was R^2^ = 0.866; based on Theils’ statistic, the deviation from the unexplained variance was Uerror = 86%, whereas the percentage of error associated with model bias and deviation from the 1:1 line was Ubias = 2% and Uslope = 12%, respectively.

## 4 Discussion

In this research, we developed a general, weather-driven, mechanistic model for the prediction of anthracnose diseases caused by *Colletotrichum* spp. on aerial plant parts of different hosts in the field. Previous models for anthracnose diseases were species- and crop-specific and considered only one component of the pathogen life cycle, mainly conidial infection (Dodd et al., 1991; Park et al., 1992; Monroe et al., 1997; Moral et al., 2012; Singh, 2020). To the best of our knowledge, this is the first time that this kind of model has been developed, calibrated for different clades and host plants, and evaluated to make predictions of anthracnose patterns on multiple crops.

Our model is “general” in that it has one conceptual structure that incorporates the key epidemiological components of anthracnose diseases. Even though *Colletotrichum* spp. exhibit numerous lifestyles–which have been categorized as necrotrophic, hemibiotrophic, latent or quiescent, and endophytic, with hemibiotrophic being the most common (Peres et al., 2005; De Silva et al., 2017)–all of the species have a necrotrophic stage (Prusky et al., 2013), except for the few species that live entirely as endophytes (e.g., some species in the gloeosporioides clade on *Salacia* and *Camellia*; Bhagya et al., 2011; Liu et al., 2015). The timing of the switch from biotrophy to necrotrophy depends on the host, its growth stage, and environmental conditions (Wharton et al., 2001; Peres et al., 2005), as well as the phylogenetic clade (De Silva et al., 2017). For instance, graminicola and destructivum clades have a short biotrophic period, while acutatum and gloeosporioides clades have a quiescent lifestyle on some fruit trees like almond and guava (De Silva et al., 2017). Our model accounts for the common attributes of the *Colletotrichum* lifestyles in terms of (i) a reproductive (asexual) stage, which occurs on the host plant or in plant debris; (ii) infection caused by conidia; (iii) an asymptomatic (biotrophic) stage that can be short or extended (depending on an incubation period); (iv) a necrotrophic stage in which host cells are invaded and killed, with the appearance of symptoms (lesions in our model); and (v) a sporulation stage, i.e., the production of secondary inoculum on lesions.

Our model is weather driven in that it accounts for the effects of temperature, wetness duration, and rain on the epidemiological components leading to disease development. The model works with an hourly time step to better account for fluctuations in temperature, as well as in wetness duration and wetness interruption that directly influence the epidemiological processes. This ensures more accurate and robust predictions than provided by models that work with a daily time step (Scherm and Van Bruggen, 1994; Narouei-Khandan et al., 2020). To be incorporated into our model, however, new information must have an hourly time step.

To design and calibrate the model, we performed a systematic literature search in order to collect published information on the biological processes involved in the development of epidemics and the weather conditions affecting these processes. Locating and assembling published knowledge by means of a systematic approach reduces errors, limits search bias, and improves the synthesis of research findings (Candel, 2014; Scherm et al., 2014), facilitating the application of system analysis to the collected information (Rossi et al., 2015). Organization of the available knowledge on the basis of system analysis revealed incomplete information for some biological processes or for some clades (especially the dematium and graminicola clades); to deal with this incomplete information, we made simplifications, made explicit assumptions, and/or used data from related clades for both model design and calibration.

The first simplification in the design of our model concerns the role of sexual spores in the *Colletotrichum* life cycle. Sexual fruiting bodies (perithecia) can be produced on artificial media by species in the gloeosporioides, destructivum, and graminicola caldes (De Silva et al., 2017). Perithecia, however, rarely occur in the field (Dowling et al., 2020), and the asexual stage is considered the sole or main kind of inoculum in disease development. The model therefore focuses on the asexual stage of *Colletotrichum*, and asexual spores (conidia) produced in acervuli are considered as the only inoculum responsible for the development of anthracnose epidemics. Exclusion of a possible (even though unlikely) ascosporic inoculum could lead to an underestimation of the total inoculum dose in the first phase of epidemics (the so-called lag phase; Schein, 1963), so delaying the epidemic growth (Schein, 1963) especially in rainless periods, because ascospores are forcibly discharged from perithecia even in the absence of rain if there is sufficient humidity (Aylor and Anagnostakis, 1991; Manstretta and Rossi, 2016).

A second simplification concerns the model’s structure. The model is based on the well-established framework designed by Zadoks (1971), in which host sites go from healthy, to infected with latent infection, to infected with visible lesions, to infectious, and finally to removed (sites with sterile lesions). This structure has been successfully applied to many pathosystems, under different climates, and on cereals and dicotyledon crops (Djurle and Yuen, 1991; Rossi et al., 1997; Savary et al., 2015; Bove et al., 2020). This model structure assumes that all sites have equal size, that healthy sites have an equal probability to become diseased, and that diseased sites are randomly distributed. For those anthracnose diseases in which lesions enlarge, such as occurs on apple (Grammen et al., 2019; Nita et al., 2019) and strawberry (Garrido et al., 2008), the assumption that lesions are equal in size could lead to an underestimation of both disease severity and the dose of secondary conidia; a lesion expansion component (Berger et al., 1997) could be easily integrated into the model for those cases. Given that *Colletotrichum* conidia are splash-borne, with short flight distances of splash droplets from the source, the assumption that diseased sites are randomly distributed is likely to be wrong, i.e., diseased sites are likely to be aggregated in space (Madden, 1997); therefore, the assumptions that all sites have an equal probability to become affected could result in an overestimation of disease severity. The introduction of a disease-aggregation coefficient for the calculation of infection rate could account for the spatial heterogeneity in disease distribution (Waggoner and Rich, 1981).

A third simplification of model design refers to the host plant. The model does not consider host growth and senescence, and only uses the growth stage to determine the periods in which plants are susceptible to infection or not. Crop growth and senescence cause changes over time of the leaf area and, consequently, of healthy sites (Bove et al., 2020). Our simplification could therefore lead to an overestimation of healthy sites in the early season when the canopy is growing, or in late season when sites become senescent, both of which could both result in an overestimation of the disease severity. As before, the model structure could be modified by inserting components for host growth and senescence (Savary et al., 2015; Bove et al., 2020). That the host can be either susceptible or not is also a simplification in our model, because there are cases in which fruits show various levels of susceptibility to infection, and in which the switch from biotrophy to necrotrophy depends on physiological and biochemical changes during the fruit ripening process (Moral et al., 2009; Moral et al., 2012; Prusky et al., 2013; Nekoduka et al., 2018; López-Moral et al., 2019).

The model was calibrated for seven major clades (i.e., acutatum, dematium, destructivum, gloeosporioides, graminicola, and orbiculare) and the singleton species *C. coccodes* (considered as the coccodes clade in the current research). Calibration was done at the clade level because of the scarcity of information at the species level; when there was no information for a specific clade, the model was operated by using the parameterization of other clades. This was the case for the effect of wetness on sporulation from primary sources only for the acutatum clade on olive and strawberry (Leandro et al., 2003; Moral and Trapero, 2012), and for the seasonal availability of primary inoculum only for the acutatum clade on olive (Moral and Trapero, 2012) only. Further studies are needed to clarify the effect of wetness on primary inoculum production and on the longevity of primary inoculum sources. A lack of information was also found for the effect of wetness on infection by the graminicola clade and the effect of temperature on incubation and latency periods for the dematium clade. In the validation, the model was operated by using equations from the closest related clade, i.e., by using equations from the destructivum clade for the graminicola clade and from the acutatum clade for the dematium clade (Salotti et al., 2022).

A clade-based calibration has some limitations. For instance, the acutatum clade, which affects strawberry, almond, olive, lupin, etc., showed great variability in incubation and latency periods depending on the host. At 15°C, the latency period was 5 days on strawberry fruits (King et al., 1997), 19 days on olive fruits (Moral et al., 2012), and about 10 days on lupin (Diggle et al., 2002). Because of this variability, the equations developed for such polyphagous clades had generally lower CCC values, higher RMSE values, and CRM values more distant from zero (**Table 4**). Effects of high within-clade variability were further reflected in the model validation results (**Table 5**), which showed that predictions were better for clades that had a small number of species and hosts. The acutatum and gloeosporioides clades, which include multiple species and hosts, had overall CCC values of 0.895 and 0.910, respectively. In contrast, the graminicola clade, which is a well-defined monophyletic clade encompassing *Colletotrichum* species mainly associated with grasses (Talhinhas and Baroncelli, 2021), had the highest CCC value of 0.973.

Clade-based calibration may also have advantages. Anthracnose diseases have often been attributed to several *Colletotrichum* species belonging to the same clade, depending on the region. For instance, the main causal agents of olive anthracnose are in the acutatum clade, with *C. godetiae* dominant in Spain, Italy, Greece, and Tunisia; with *C. nymphaeae* dominant in Portugal; and with *C. fioriniae* dominant in California (Moral et al., 2021). In addition, proper identification of the *Colletotrichum* species requires molecular analysis of multiple DNA regions (Damm et al., 2009; Damm et al., 2012; Weir et al., 2012; Damm et al., 2013; Damm et al., 2014). Because the species belonging to the same clade show similar environmental requirements (Salotti et al., 2022), a clade-based calibration may be useful to overcome knowledge gaps for single *Colletotrichum* species, to favor practical model usage when the identification of species is not possible because of capacity or economic limitations, and to make predictions for a wide range of hosts and climatic conditions.

Given that the model was validated against independent data (i.e., data not used in model development) and provided accurate and robust predictions of anthracnose epidemics, we conclude that our assumptions and simplifications did not greatly reduce the model’s ability to make correct predictions. Overall, high concordance was shown between model predictions and reality (CCC = 0.928), with few errors (RMSE = 0.044), and a slight tendency of the model toward overestimation (CRM = –0.052). Unfortunately, data enabling model validation were available for only five of the eight clades for which the model was parameterized (i.e., acutatum, dematium, gloeosporioides, graminicola, and orbiculare clades). Except for the acutatum clade, which was validated on strawberry and olive, each clade was validated on only one host. More complete validation is needed for different clades and hosts.

In our model validation, the *k* dose of primary conidia that can develop from overwintering inoculum sources (crop debris, mummies, and dormant buds) was estimated empirically from disease data; this estimation may not greatly affect the reliability of the model to predict disease progress because it modulates the final value of the disease severity but not its progress. The real value of *k* in a field may depend on many difficult-to-estimate factors, including the incidence and severity of affected crops in the previous season and the proportion of diseased tissues that remains in the field as an overwintering inoculum source. Inappropriate estimation of *k* may result in under- or overestimation of infection risk during the season. Further studies are therefore needed to improve the estimation of the abundance of primary inoculum sources at the beginning of the cropping season.

Our model is flexible in both design and calibration. For instance, the model can easily incorporate components for lesion expansion, plant growth, senescence, or host susceptibility by integration of additional model components (Loomis and Adams, 1983) and of modifiers accounting for resistance components, as has been done with similar models (Savary et al., 2015; Bove et al., 2021). Model flexibility also enables easy incorporation of new information on pathogen biology and epidemiology, so that inserting species-specific or host-specific calibrations may improve the prediction accuracy especially in hosts or in regions in which anthracnose disease is caused by a dominant *Colletotrichum* species, or for clades not considered in this work such as the boniense clade, which is economically important on several *Citrus* spp. (Uysal and Kurt, 2019; Talhinhas and Baroncelli, 2021).

In spite of some shortcomings mainly related to its simplicity and the scarcity of information available in the literature, our model is promising. After further validation and evaluation of its ability to support risk-based fungicide applications, the model could be used for supporting decision-making in crop protection.

## Data availability statement

The original contributions presented in the study are included in the article/**Supplementary Material**. Further inquiries can be directed to the corresponding author.

## Author contributions

IS, Y-JL, and VR mainly contributed to the conceptualization of the model. VR provided the methodology and the resources for the study. IS, Y-JL, and TJ performed model validation. All authors contributed to the article and approved the submitted version.
