# Supplementary material for: Development of a model for Colletotrichum diseases with calibration for phylogenetic clades on different host plants
Source: Front Plant Sci. 2023 Mar 29;14:1069092. doi: 10.3389/fpls.2023.1069092 (PMC10090521; doi:10.3389/fpls.2023.1069092)
Supplement: Supplementary file 1 [file DataSheet_1.pdf]

Supplementary Material

This supplementary material describes the search strings used in the literature search (Table S1) and the predicted and observed disease progress curves for epidemics that are listed in Table 2 but are not included in the manuscript (Figures S1–S5).

Supplementary Table 1. Search strings for biological processes considered for the literature search in three databases and the corresponding number of papers found.

Process	Database	Keywords	Number of hits
Primary inoculum production	Scopus	TITLE-ABS-KEY ((Colletotrichum)AND(“primary inoculum” OR conidia OR spore* OR acervuli*)AND(production OR development OR overwinter*)AND(temperature* OR wetness OR “relative humidity” OR environment*))	177
	Web of science	TS=((Colletotrichum)AND(“primary inoculum” OR conidia OR spore* OR acervuli*)AND(production OR development OR overwinter*)AND(temperature* OR wetness OR “relative humidity” OR environment*))	150
	CAB Abstract	(Colletotrichum and primary inoculum and production).af.	124
Dispersion of conidia	Scopus	TITLE-ABS-KEY ((Colletotrichum)AND(conidia OR spore*)AND(dissemination OR release OR dispers* OR deposition OR rain))	87
	Web of science	TS=((Colletotrichum)AND(conidia OR spore*)AND(dissemination OR release OR dispers* OR deposition OR rain))	115
	CAB Abstract	(Colletotrichum and conidia and dispersal).af.	17
Infection of conidia	Scopus	TITLE-ABS-KEY ((Colletotrichum)AND(conidia OR spore*)AND(infection OR appressori* OR penetration OR germination)AND(wetness))	18
	Web of science	TS=((Colletotrichum)AND(conidia OR spore*)AND(infection OR appressori* OR penetration OR germination)AND(wetness))	24
	CAB Abstract	(Colletotrichum and infection and wetness).af.	57
Survival of conidia	Scopus	TITLE-ABS-KEY ((Colletotrichum)AND(conidia OR spore*)AND(survival)AND(temperature* OR wetness OR “relative humidity” OR environment*))	18
	Web of science	TS=((Colletotrichum)AND(conidia OR spore*)AND(survival)AND(temperature* OR wetness OR “relative humidity” OR environment*))	32
	CAB Abstract	(Colletotrichum and conidia and survival).af.	10
Incubation period	Scopus	TITLE-ABS-KEY ((Colletotrichum)AND(incubation OR “lesion development” OR “lesion onset”)AND(temperature* OR “relative humidity” OR environment*))	109
	Web of science	TS=((Colletotrichum)AND(incubation OR “lesion development” OR “lesion onset”)AND(temperature* OR “relative humidity” OR environment*))	82
	CAB Abstract	(Colletotrichum and incubation).af.	123
Latent period	Scopus	TITLE-ABS-KEY ((Colletotrichum)AND(latency OR “latent period”)AND(temperature* OR “relative humidity” OR environment*))	14

	Web of science	TS=((Colletotrichum)AND(latency OR “latent period”)AND(temperature* OR “relative humidity” OR environment*))	18
	CAB Abstract	(Colletotrichum and latency).af.	11

The operator AND indicates that both terms must be present somewhere in the search field; the operator OR indicates that at least one term must be present in the search field. The high quotes (“”) allow more than one word to be considered as a single item. The wildcard (*) allows the selection of multiple word endings.

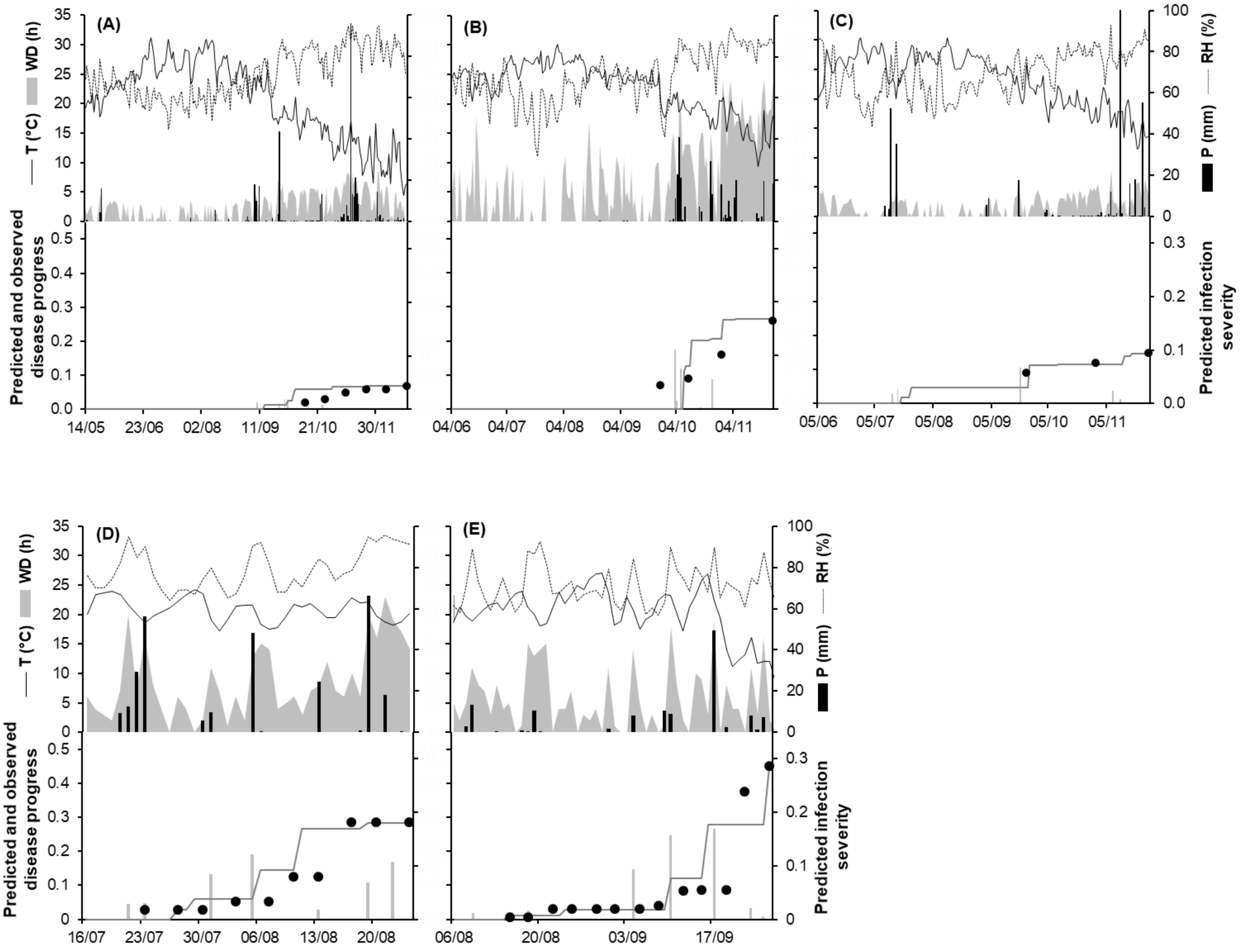

Supplementary Figure 1. Predicted and observed disease progress on olive (A-C) and strawberry (D-E) for the acutatum clade in (A) Nardò, Apulia, Italy, in 2017 (IT-17B); (B) Avetrana, Apulia, Italy, in 2018 (IT-18); (C) Avetrana, Apulia, Italy, in 2019 (IT-19); (D) Wooster, Ohio, USA, in 1990 (OH-90); and (E) Wooster, Ohio, USA, in 1991 (OH-91). *Upper panels*: Weather variables, including air temperature (T, °C, solid line), relative humidity (RH, %, dotted line), rainfall (P, mm, black bars), and wetness duration (WD, in h, gray area); *Lower panels*: Infection severity predicted by the model (light gray bars), disease severity predicted by the model (dark gray line), and observed disease incidence (full dots).

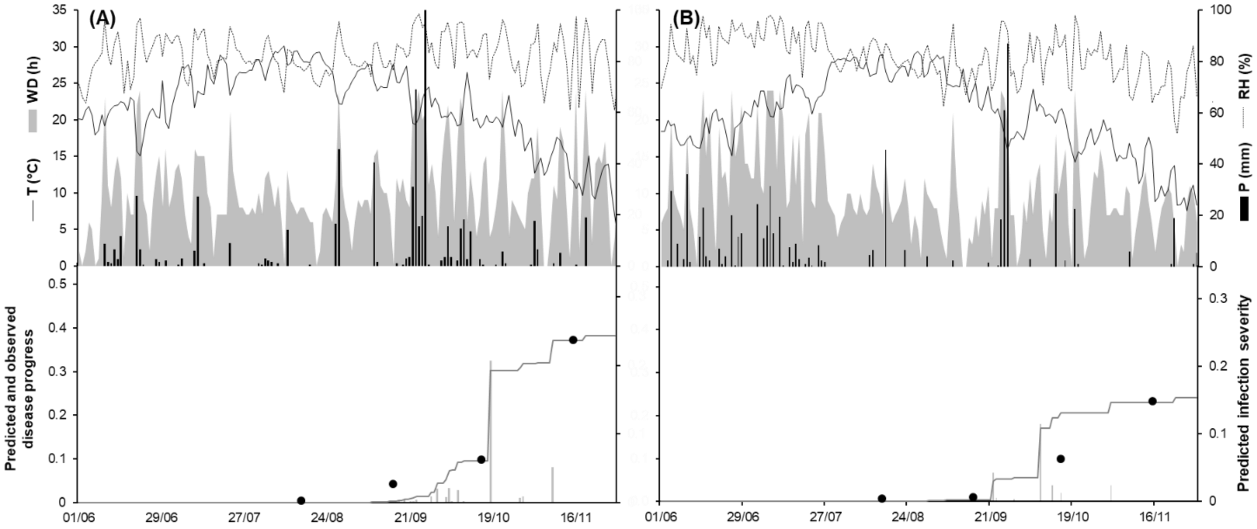

Supplementary Figure 2. Predicted and observed disease progress on mulberry for the dematium clade in (A) Tsukuba, Ibaraki, Japan, in 1994 (JA-94) and (B) Tsukuba, Ibaraki, Japan, in 1995 (JA-95). *Upper panels*: Weather variables, including air temperature (T, °C, solid line), relative humidity (RH, %, dotted line), rainfall (P, mm, black bars), and wetness duration (WD, in h, gray area). *Lower panels*: Infection severity predicted by the model (light gray bars), disease severity predicted by the model (dark gray line), and observed disease incidence (full dots).

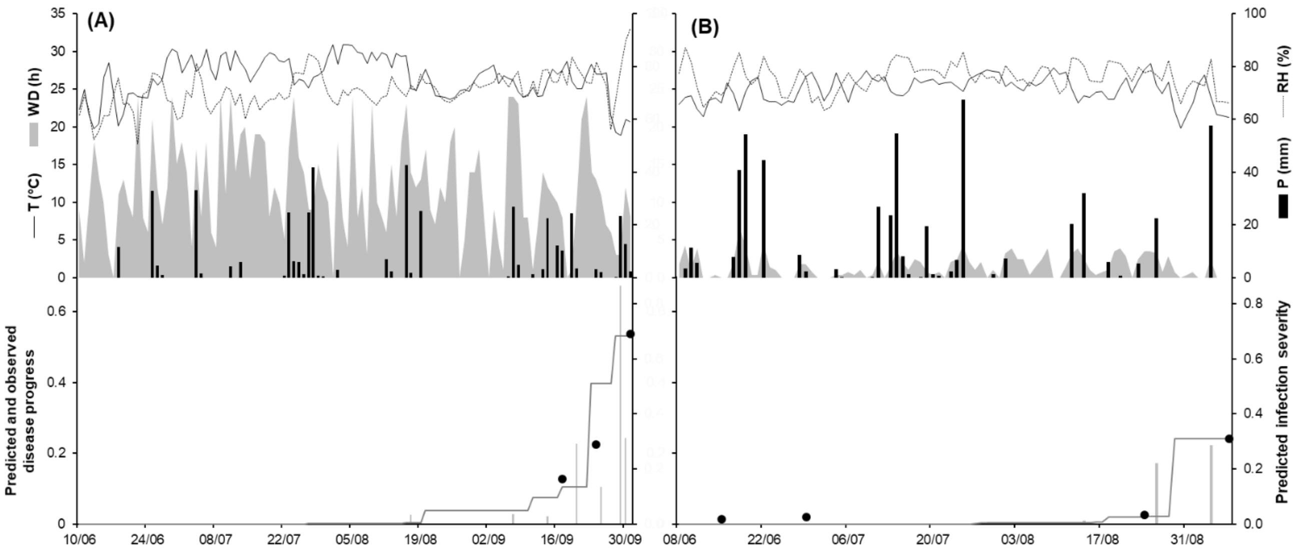

Supplementary Figure 3. Predicted and observed disease progress on grape for the gloeosporioides clade in (A) Castle Hayne, North Carolina, USA, in 1980 (NC-80) and (B) Castle Hayne, North Carolina, USA, in 1982 (NC-82). *Upper panels*: Weather variables, including air temperature (T, °C, solid line), relative humidity (RH, %, dotted line), rainfall (P, mm, black bars), and wetness duration (WD, in h, gray area). *Lower panels*: Infection severity predicted by the model (light gray bars), disease severity predicted by the model (dark gray line), and observed disease incidence (full dots).

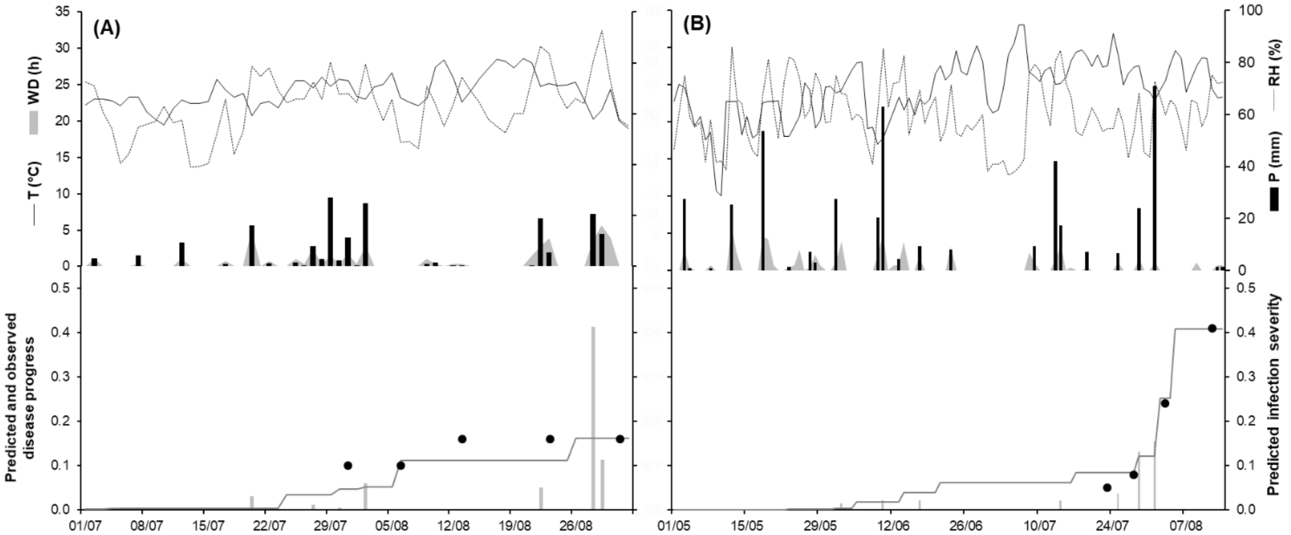

Supplementary Figure 4. Predicted and observed disease progress on bluegrass for the graminicola clade in (A) North Brunswick, New Jersey, USA, in 2009 (NJ-09) and (B) North Brunswick, New Jersey, USA, in 2010 (NJ-10). *Upper panels*: Weather variables, including air temperature (T, °C, solid line), relative humidity (RH, %, dotted line), rainfall (P, mm, black bars), and wetness duration (WD, in h, gray area). *Lower panels*: Infection severity predicted by the model (light gray bars), disease severity predicted by the model (dark gray line), and observed disease incidence (full dots).

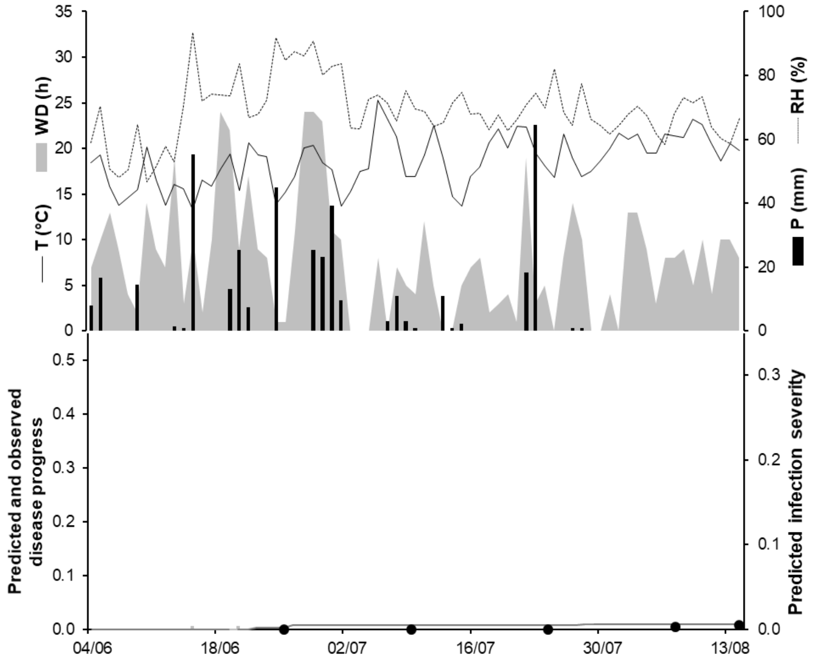

Supplementary Figure 5. Predicted and observed disease progress on dry bean for the orbiculare clade in Morden, Manitoba, Canada, in 2014 (MA-14). *Upper panel*: Weather variables, including air temperature (T, °C, solid line), relative humidity (RH, %, dotted line), rainfall (P, mm, black bars), and wetness duration (WD, in h, gray area). *Lower panel*: Infection severity predicted by the model (light gray bars), disease severity predicted by the model (dark gray line), and observed disease incidence (full dots).
